# Somatotropic Axis Regulation Unravels the Differential Effects of Nutritional and Environmental Factors in Growth Performance of Marine Farmed Fishes

**DOI:** 10.3389/fendo.2018.00687

**Published:** 2018-11-27

**Authors:** Jaume Pérez-Sánchez, Paula Simó-Mirabet, Fernando Naya-Català, Juan Antonio Martos-Sitcha, Erick Perera, Azucena Bermejo-Nogales, Laura Benedito-Palos, Josep Alvar Calduch-Giner

**Affiliations:** Nutrigenomics and Fish Growth Endocrinology, Institute of Aquaculture Torre de la Sal (IATS-CSIC), Castellón, Spain

**Keywords:** growth hormone, insulin-like growth factors, insulin-like growth factor binding proteins, growth hormone receptors, insulin and IGF receptors, sirtuins, oxygen availability, energy status

## Abstract

The Gh/Prl/Sl family has evolved differentially through evolution, resulting in varying relationships between the somatotropic axis and growth rates within and across fish species. This is due to a wide range of endogenous and exogenous factors that make this association variable throughout season and life cycle, and the present minireview aims to better define the nutritional and environmental regulation of the endocrine growth cascade over precisely defined groups of fishes, focusing on Mediterranean farmed fishes. As a result, circulating Gh and Igf-i are revitalized as reliable growth markers, with a close association with growth rates of gilthead sea bream juveniles with deficiency signs in both macro- or micro-nutrients. This, together with other regulated responses, promotes the use of Gh and Igf-i as key performance indicators of growth, aerobic scope, and nutritional condition in gilthead sea bream. Moreover, the sirtuin-energy sensors might modulate the growth-promoting action of somatotropic axis. In this scenario, transcripts of *igf-i* and *gh* receptors mirror changes in plasma Gh and Igf-i levels, with the *ghr-i/ghr-ii* expression ratio mostly unaltered over season. However, this ratio is nutritionally regulated, and enriched plant-based diets or diets with specific nutrient deficiencies downregulate hepatic *ghr-i*, decreasing the *ghr-i*/*ghr-ii* ratio. The same trend, due to a *ghr-ii* increase, is found in skeletal muscle, whereas impaired growth during overwintering is related to increase in the *ghr-i*/*ghr-ii* and *igf-ii*/*igf-i* ratios in liver and skeletal muscle, respectively. Overall, expression of insulin receptors and *igf* receptors is less regulated, though the expression quotient is especially high in the liver and muscle of sea bream. Nutritional and environmental regulation of the full Igf binding protein 1–6 repertoire remains to be understood. However, tissue-specific expression profiling highlights an enhanced and nutritionally regulated expression of the *igfbp-1/-2/-4* clade in liver, whereas the *igfbp-3/-5/-6* clade is overexpressed and regulated in skeletal muscle. The somatotropic axis is, therefore, highly informative of a wide-range of growth-disturbing and stressful stimuli, and multivariate analysis supports its use as a reliable toolset for the assessment of growth potentiality and nutrient deficiencies and requirements, especially in combination with selected panels of other nutritionally regulated metabolic biomarkers.

## Expansion of Gh/Prl/Sl family

The fish growth hormone (Gh) and prolactin (Prl) family was initially expanded with the discovery of somatolactin (Sl) in olive flounder (1) and Atlantic cod (2). Thereafter, Sl has been identified, purified or recombinantly produced from a multitude of teleost species, including chum salmon (3), sole (4, 5), gilthead sea bream (6, 7), goldfish (8), eel (9), rainbow trout (10), and European sea bass (11, 12). Sl has also been identified in primitive fishes, such as the white sturgeon and the West African lungfish (13), which suggests that the ancestors of Gh and Sl were present before the divergence of lobe-finned fishes (*Sarcopteygii*) and ray finned fishes (*Actinopterygii*). Thereafter, the *sl* gene was duplicated in the basal teleost tetraploidization (3R), giving rise to s*l*α and s*l*β, which were first identified in zebrafish (14) and Atlantic salmon (15). However, the *sl*β gene was lost from the lineage, leading to spiny-rayed fishes (*Acanthomorpha*), so it is not found in the most diverse and species-rich group of modern teleosts (16). Conversely, Prl2, the last member of the fish Gh/Prl/Sl family, has been identified in almost all non-mammalian vertebrate species ranging from cartilaginous fishes to tetrapods (17, 18). Despite this, both the *sl* and *prl2* genes were lost twice independently in tetrapods: once at the base of the amphibian lineage and once early in mammalian evolution (16, 19).

**Figure 1:**
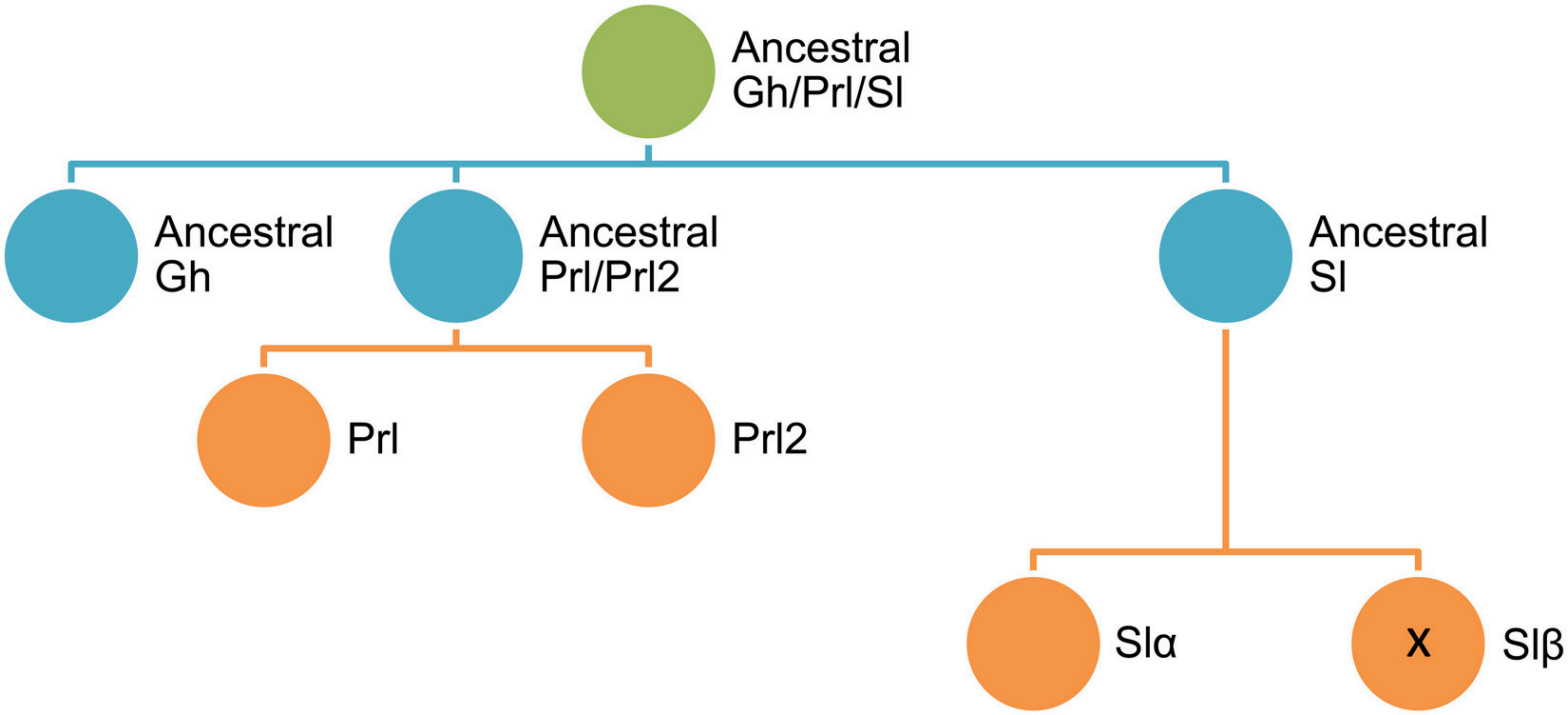
Schematic representation of the diversification of Gh/Prl/Sl family in fishes.

At a closer look, proteins of the Gh/Prl/Sl family share a common genomic organization with five to six exons in almost all fish species examined, including cyprinids, salmonids, flat fishes and Perciformes (20–22). Another characteristic feature is the cysteine residues involved in disulfide bridges, one linking distant parts of the polypeptide chain and another forming a loop close to the C-terminus that are strictly retained through vertebrate evolution. However, an additional N-terminal disulphide loop appeared in the Sl and Prl/Prl2 branches, prior to the divergence of bony fishes from the lineage leading to tetrapods, and thereafter the specific *sl* and *prl2* deletion events occurred between lungfish and amphibian lineages (23). In this context, the precise timing of gene losses and early duplication events is difficult to establish, though analyses of phylogeny and conserved synteny suggest that the *gh* and *prl/prl2* genes arose likely from a local duplication before 1R tetraploidization, and a second local gene duplication within the same time window gave rise to *sl* (16, 24) (see Figure 1).

Synteny approaches also reveal that the Gh receptors (Ghr) and Prl receptors (Prlr) are located on the same chromosome, probably as the result of an early local gene duplication (25). By contrast, the Sl receptors (Slr), identified as belonging to the Ghr-i clade, might have arisen much later in the 3R tetraploidization. This would be an example of subfunctionalization, where the named Ghr-i and Ghr-ii might have evolved for differential ligand binding preferences for Sl or Gh, respectively. However, in contrast to the ligand binding study in masu salmon (26), subsequent studies in black sea bream (27), and zebrafish (28) did not reveal differences between Ghr-i and Ghr-ii in terms of ligand binding affinities. Moreover, Ghr-i preferentially binds Gh rather than Sl in Japanese eel (29) and trout (30) binding assays. It appears, therefore, that the observed fish species differences in Ghr binding are more likely to be due to the different natures of Gh/Sl preparations or other factors that are hitherto unknown. Moreover, Bergan-Roller and Sheridan (31) pointed out that one Ghr would be more responsive for transmitting lipolytic or stress signals of Gh/Sl, while the other Ghr subtype would be more active in transmitting growth-promoting signals. In this context, it is of relevance to define clear patterns of Ghr expression in combination with changes in other growth- and metabolic-related factors and, more importantly, how the somatotropic axis is affected as a whole by the advent of new rearing systems and diet formulations for the intensification of fish farming in the 2030 horizon. In this regard, the aim of this minireview is to update the environmentally-mediated changes in fish somatotropic axis activity, linking them with a more accurate phenotyping of fish nutritional and metabolic status through the development and productive cycles. Attention is focused on marine fishes with special emphasis on gilthead sea bream (*Sparus aurata*) as the most important farmed fish of the Mediterranean aquaculture.

## Fish Gh/Sl sub-functionalization

**Figure 2:**
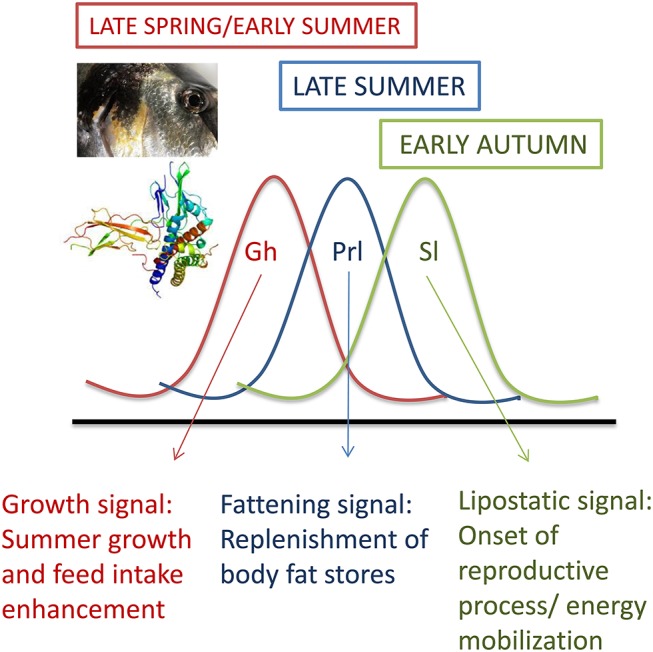
Seasonal patterns of plasma levels of Gh, Prl, and Sl in juveniles of gilthead sea bream. Adapted from Pérez-Sánchez et al. (20).

Life-history decisions are not fixed and often depend on critical size and sufficient energy at a specific stage (“opportunity window”) several months prior to biological transformations. For instance, the decisive factor in salmonids to become smolts or sexually mature is linked to growth and fat depositions at mid-summer and spring (32, 33). Thus, plasma Gh levels in most fishes, including gilthead sea bream, peak at late spring and early summer, whereas the peaks of Prl and Sl are delayed to mid-summer and autumn, respectively (20, 34). Therefore, Gh, Prl and Sl are differentially regulated on a seasonal basis, but also in response to energy availability (Figure 2). Certainly, circulating Sl increases transitorily with fasting in gilthead sea bream, but, opposite to Gh, a persistent increase in plasma Sl is found with the increase in adiposity in overfed or old animals (35, 36). This supports a close association between Sl and lipostatic signals in fishes and gilthead sea bream in particular. In agreement with this, murine leptin is able to stimulate the *in vitro* secretion of pituitary Sl as part of the nutritional mechanisms driving the onset of puberty in European sea bass (37), though the seasonal pattern of Sl is more erratic in European sea bass than in gilthead sea bream, and it is difficult to infer a characteristic Sl pattern when PIT-tagged animals are considered individually (38). In any case, studies in gilthead sea bream highlight that recombinant Sl triggers a transient inhibition of feed intake, a satiety effect that is related to decreases in the respiratory quotient (CO_2_ output per O_2_ uptake) (12, 36). This pattern of gas exchange suggests the activation of lipid catabolism, which is consistent with the Sl inhibition of the hepatic activity of acetyl-coenzyme-A carboxylase (12). In salmonids, a role of Sl in energy mobilization during reproduction, acute stress or exhaustive exercise (39–42) has also been proposed. Therefore, Sl may help to expedite growth-reproductive processes following replenishment of fat stores and/or mediate the adaptation to fasting until the lipolytic action of Gh and/or other endocrine factors is fully accomplished. Contrary to this, other authors point out that the skin-color regulation is the only definite role of Sl so far demonstrated in fishes and medaka in particular (43). This still remains under debate, but the consensus is that Sl does not enhance *in vivo* or *in vitro* the production of insulin-like growth factors (Igfs) (12, 44, 45).

## Gh-dependent growth

Pituitary cells producing GH are the main source of this hormone in the body, and its circulating concentration is barely detectable following hypophysectomy or impaired somatotroph differentiation in humans and birds (46). Hence, pituitary GH is largely responsible for the endocrine actions of GH during perinatal and postnatal growth, and according to the classical somatomedin hypothesis GH is released into the systemic circulation and then transported to target tissues to act via specific receptors to regulate growth and metabolism, directly or indirectly through the induction of hepatic or locally produced IGFs (47). However, this concept changed in the last quarter of twentieth century with the evidence of a widespread distribution of GH in most extrapituitary tissues, including neuronal, reproductive, immune, and gastrointestinal tissues, which may be independent on the pituitary-specific transcription factor-1 (PIT-1) (48). Indeed, pituitary GH expression does not occur in early embryonic or fetal development, whereas extrapituitary GH is expressed much earlier, prior to its ontogenic appearance in pituitary somatotrophs. In other words, as highlighted in previous reviews (49–52), embryonic or early fetal growth is independent of pituitary GH in higher vertebrates, and the persistence of extrapituitary GH through the life cycle is indicative of paracrine or autocrine roles for locally-produced GH. Indeed, the functional relevance of this extrapituitary Gh in postnatal growth would vary among vertebrates. For instance, the decreased expression of extrapituitary *GH* is not related to dwarf phenotypes in humans (51), whereas interactions between systemic and locally produced GH may contribute to explain the inconsistent relationship of circulating GH and growth in chickens (49).

As in birds and humans, extrapituitary Gh has been detected in immunorelevant and reproductive tissues of gilthead sea bream, trout and Japanese eel (53–55). The *gh* gene is transcribed and translated early during fish larval development before or after pituitary differentiation (29, 56, 57). Moreover, the main components of the somatotropic axis (Gh, Ghrs, Igfs) are produced as soon as transcription starts in fish embryos (58), and binding studies revealed a high concentration of actively transcribed Ghrs on the head of gilthead sea bream larvae a few days after hatching, which is consistent with their allometric growth. All this evidence together suggests a direct action of Gh rather than systemic effects via hepatic Igfs during early life stages (59), but later in life this complex trade-off would have evolved in a different manner within each group of vertebrates.

## Circulating GH and IGF-i

### Growth and IGF-I association

IGFs are the primary mediators of the growth-promoting effects of GH, operating in an autocrine, paracrine and endocrine manner (60). They affect many biological processes, including protein synthesis and turnover, cell proliferation and differentiation, and cell apoptosis and tissue maintenance, which make IGFs good candidates as growth indexes. Unlike GH and insulin, IGFs are not stored or released in pulses, and clearance rates are retarded by the action of a suite of IGF-binding proteins (IGFBPs), a feature that allows relatively constant levels of IGFs in the blood. However, as reviewed by Beckman (61), the correlation between Igf-i and growth ranges from tight to non-discernible in fishes, probably reflecting a changing and sometimes confounding scenario. Thus, ration size, circulating Igf-i and growth rates are often positively correlated (62–64), though the Igf system appears to have a notable inertia after extended fasting or feed-restriction periods.

Overall, fast-growing fish strains also share an enhanced somatotropic axis activity (65, 66). However, the relationship between Igf-i and growth is largely affected by a wide-range of endogenous and exogenous factors, including gender, developmental and maturity state, photoperiod, temperature and salinity as well as stress and disease condition, which makes the Igf-i and growth relationship variable over season and productive cycles (61). Accordingly, Igf-i is a reliable growth index over precisely defined groups of fishes, but conservative approaches are needed for comparisons of Igf-i and growth rates across experiments within and between different fish species. For instance, the true effects of different photoperiods on growth and plasma Igf-i level are difficult to discern in trout and salmon (67–69). Likewise, some seasonal delay exists between growth rates and plasma Igf-i level over the productive cycle of gilthead sea bream (34), though circulating Igf-i continues to be perceived as one of the most reliable markers of growth performance in a wide range of fishes, including the hybrid striped bass (70), juvenile lingcod (71), or Nile tilapia (72). Furthermore, a concordant Igf-i and growth relationship has been reported combining data from juveniles of three Mediterranean farmed fishes (European sea bass, gilthead sea bream, common dentex) reared in the same indoor experimental facilities (35). A relatively high degree of concordance was also found for the decreased growth rates and circulating level of Igf-i in 1-, 2-, and 3-year old gilthead sea bream (73). Likewise, the recovery of plasma Igf-i during refeeding highly reflects the increase in weight gain during the phase of compensatory growth (74). More recently, the circulating level of Igf-i was concordant with the growth-promoting effects of moderate exercise in juveniles of gilthead sea bream (75, 76). Also in gilthead sea bream, a linear increase in growth rates and circulating Igf-i was reported in fingerlings in response to a single dose of recombinant bovine GH (77). However, these growth/endocrine patterns highly differ from those found in salmonids and, for the same or even higher growth rates, the circulating concentrations of Gh and Igf-i are often 3–10 times lower in trout juveniles than in Mediterranean farmed fishes (78).

Fish-species differences in the regulation of the Gh/Igf system also apply to the osmoregulatory action of Gh, which may act synergistically with cortisol and Igf-i to enhance the hypo-osmoregulatory ability, mainly in salmonid species. Thus, anadromous migrations from rivers to hyperosmotic environments stimulates the somatotropic axis for rapid growth and triggers different osmoregulatory actions related to the development of preparatory mechanisms for seawater entry (79). In this regard, Gh action can be mediated by Ghrs to stimulate Igf-i production not only in liver, but also in osmoregulatory organs (gills, kidney, and intestine), which in turn orchestrates ion and water movements to preserve or achieve a new steady state of plasma osmolality, as reported in Sakamoto et al. (80). However, confounding results have been reported in non-salmonid species. In several tilapia species (*Oreochromis mossambicus* and *O. niloticus*), no significant differences have been reported in pituitary *gh* expression or in Gh plasma levels in juvenile or larval stages in freshwater-, brackishwater- or seawater-acclimated fishes (81–83), whereas other studies suggest a role of Gh during osmotic acclimation as a result of modifications in plasma Gh level after hyperosmotic transfer (84–86). In eels, no changes in plasma Gh have been reported with transfer from freshwater to seawater (87, 88). In meager and gilthead sea bream, a clear increase in pituitary *gh* transcripts is found in hyperosmotic environments (89, 90). This response is maintained during acclimation to isosmotic salinity in silver sea bream (91, 92), black sea bream (93) or gilthead sea bream (94), which in turn would trigger the improvement of growth rate (95) through changes in the pentose phosphate pathway or synthesis of stress proteins (91, 93, 96). The ultimate mechanisms remain to be established, but importantly, microarray gene expression profiling of liver, gills and hypothalamus after hypo- or hyperosmotic challenges identified more than 750 differentially expressed genes in gilthead sea bream, with three major clusters of overlapping canonical pathways corresponding to energy metabolism, oxidative stress and cell and tissue architecture (97).

### Sirtuin energy-sensing and GH/IGF-I

Differences in key performance indicators necessarily reflect different uses of nutrients and energy, as voluntary feed intake and growth are limited by the capacity to preserve the redox balance (98–100). In that sense, animals with enhanced feed intake and growth rates are able to grow efficiently in a cellular milieu with enhanced risk of oxidative stress, and the differential regulation of sirtuins (SIRTs) contributes to the readjustment and preservation of metabolic homeostasis. These enzyme deacetylases use NAD^+^ as cofactor and couple the acetylation status of histone and non-histone substrates with the energy status of the cell via NAD^+^/NADH ratio. SIRTs are virtually ubiquitous through all kingdoms of life, and the number of family members increases with the organismal complexity: prokaryotes have one to two family members, fission yeast three, worms four, flies five and higher vertebrates (including fishes) seven (101, 102). This, together with different cellular locations (nuclear, SIRT1 and SIRT6-7; cytoplasmic, SIRT2; mitochondrial, SIRT3-5) offers the possibility of complementary but also non-redundant and tissue-specific energy-sensing mechanisms strongly influenced by nutrient availability, energy demand and tissue-specific metabolic capabilities (103–105).

The ultimate mechanisms by which changes in *SIRT* expression and activity modulate the action of metabolic hormones and the GH/IGF system remains poorly studied. However, studies in mice have found that *in vivo* knockdown of hepatic *Sirt1* restores the fasting-induced decrease in serum IGF-I and enhances the GH-dependent increase in IGF-I (106). Knockdown of *Sirt1* in mice enhances the acetylation and GH-induced tyrosine phosphorylation of STAT5, indicating that SIRT1 negatively regulates GH-dependent IGF-I production via deacetylation of transcription factor STAT5. Additionally, SIRT1 acts at the brain level as a link between somatotropic signaling and calorie restriction (107), and brain-specific *Sirt1* knockout mice have dwarfism and reduced plasma GH and IGF-I (108), displaying similar phenotypes to those of long-lived mutant mice (109). By contrast, SIRT1 activation with resveratrol suppresses GH synthesis in pituitary rat cells by reducing PIT-1 availability to the *Gh* promoter via the transcriptional suppression of *Creb* (110). Unlike this apparent controversy, the net effect of SIRT1 activation continues to be the suppression of the GH/IGF tonus, which would serve to drive a decreased energy demand for growth purposes in a cellular milieu with a reduced availability of metabolic fuels.

Relatively less is known about the regulation of *sirts* in fish, but recent studies in gilthead sea bream highlight that short-term fasting does not alter significantly the *sirt1* gene expression in liver, whereas the expression pattern for other *sirt* isotypes (*sirt2*-*6*) is an overall suppression linked to reduced energy demand for hepatic lipogenesis (102, 111). In contrast, gilthead sea bream strains that regularly perform better than other genetically different strains have a reduced hepatic *sirt1* expression, in combination with a more active feeding behavior and Gh/Igf-i system, resulting in enhanced growth and higher circulating Igf-i (112). The expression of *sirt* genes is also highly regulated by energy demand in the white skeletal muscle of gilthead sea bream, and *sirt2* mRNA is clearly upregulated in fast growing fish, whereas the mitochondrial *sirt5* emerges as a more responsive element during forced fasting (102) or natural fasting when comparisons are made between fishes of different size during the cold season (unpublished results). In both cases, this occurs in combination with the enhancement of the lipolytic machinery and reduced energy wastage, evidenced by the downregulation of muscle mitochondrial uncoupling proteins and changes in the expression of many markers of lipid metabolism and oxidative phosphorylation, as was also highlighted by the muscle microarray gene expression profiling of fishes fed at maintenance ratio (113).

**Figure 3:**
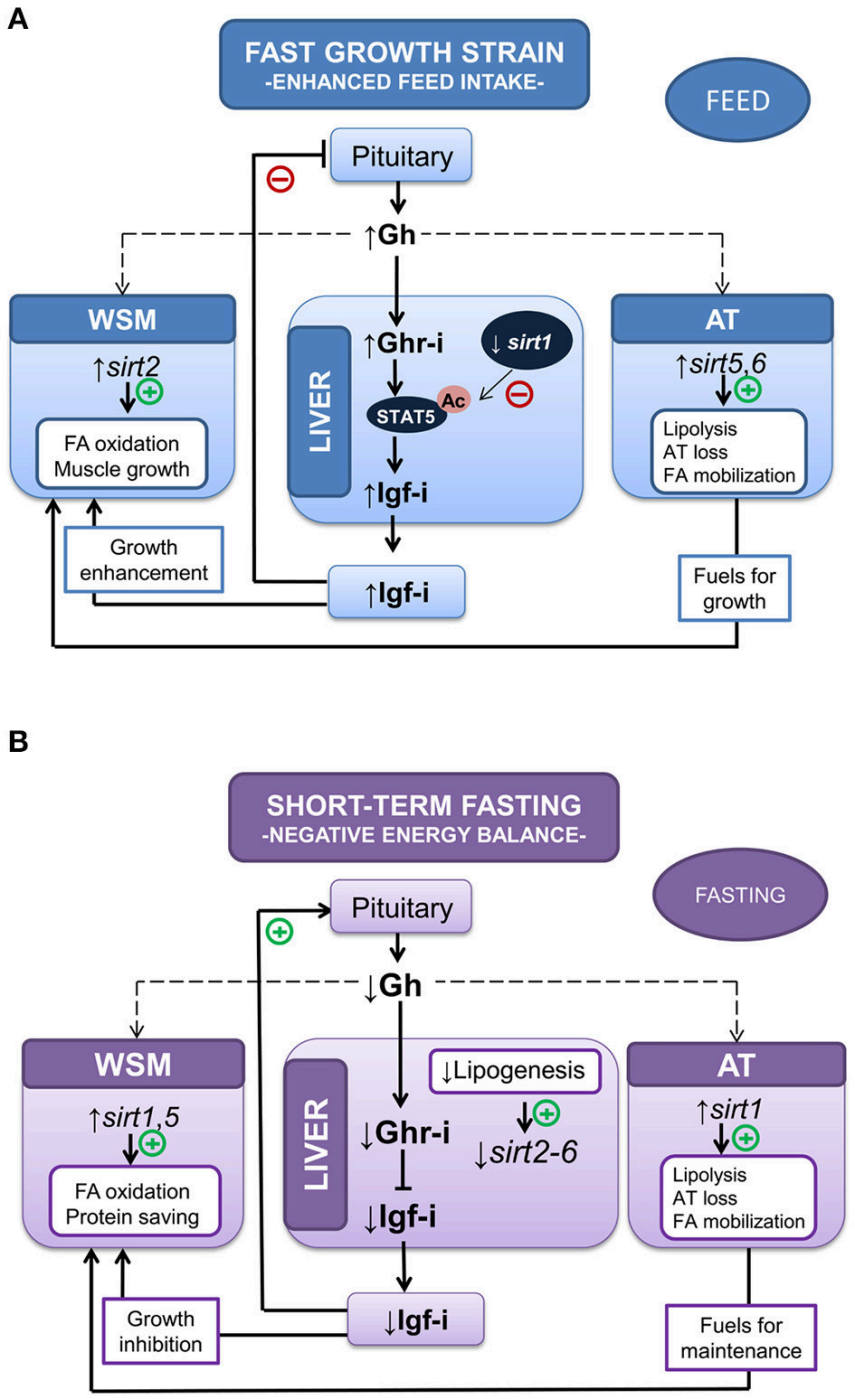
Metabolic crosstalk between Sirtuin-energy sensors and the Gh/Igf system in liver, white skeletal muscle (WSM) and adipose tissue (AT) in two different experimental models: **(A)** accelerated growth in fishes with enhanced feed and **(B)** negative energy balance (10-day-fasted fishes). Adapted from Simó-Mirabet et al. (102, 112).

For comparative purposes, it is relevant that physiological studies in humans have revealed a regulatory role of SIRT2 in muscle stem cell proliferation and differentiation (114–116). Moreover, single-nucleotide polymorphisms of *Sirt2* have been associated with different body size traits in Qinchuan cattle (117). Adiposity is also a main factor affecting *SIRT* expression, and SIRT6 activity is depressed in the adipose tissue of obese human patients (118). In this way, it is likely that the concurrent upregulation of *sirt5* and *sirt6* in the adipose tissue of our fast-growing fish strains orchestrate a lean phenotype, resulting in a reduced adipose tissue mass with an increased mobilization of fatty acids toward skeletal muscle and liver. All of the above evidences a complex metabolic crosstalk between Sirt-energy sensors and the Gh/Igf system, with perhaps the double aim of (i) avoiding or minimizing the loss of muscle protein mass during stages of negative energy balance and (ii) precisely tuning the growth energy-demanding processes of organisms to the exogenous supply and availability of metabolic fuels (see Figure 3). However, we are far from fully understand this complex picture, and current studies aiming to highlight whether tissue-specific differences in fish Sirt profile result from nutritional, genetic or epigenetic sources of variation as key players of genome stability during environmental stimuli and stress response are underway, as reviewed by Bosch-Presegué and Vaquero (119).

### Gh/Igf responsiveness in hypoxic and crowded fishes

Animals overexpressing *Gh* combat oxidative stress less efficiently than normal and dwarf mice (120, 121). In the same way, *gh*-transgenic fishes have a lower capacity to manage a hypoxic environment efficiently (122, 123), though paradoxically early studies in mammals indicate that circulating Gh can be increased by either the increase in O_2_ requirements or the reduction of O_2_ availability (124). A possible explanation is that regulation of circulating Gh level in hypoxic animals mirrors the changing energy needs rather than the availability of metabolic fuels. In this way, circulating Gh increased markedly in salmonids submitted to maximum swimming (125, 126). The same response has been found in gilthead sea bream, and plasma Gh levels are of predictive value of the aerobic scope in swimming test chambers, as circulating Gh before exercise is a surrogate marker of critical speed (swimming speed that could theoretically be maintained indefinitely without exhaustion) (127). The opposite is also true, and circulating Gh is lowered after acute or chronic confinement exposure in a wide range of fishes, including gilthead sea bream (128–130), trout (131), Atlantic salmon (132), and Nile tilapia (133). However, as first pointed out by Pickering et al. (131), hypoxia, crowding and water quality induce confounding effects that include the activation of the hypothalamic-pituitary-interrenal axis (HPI-axis) and the overall downregulation of hepatic *igf* and *ghr* genes, concomitant with growth inhibition and lowered plasma Igf-i in both crowded and hypoxic fishes (130, 134).

**Figure 4:**
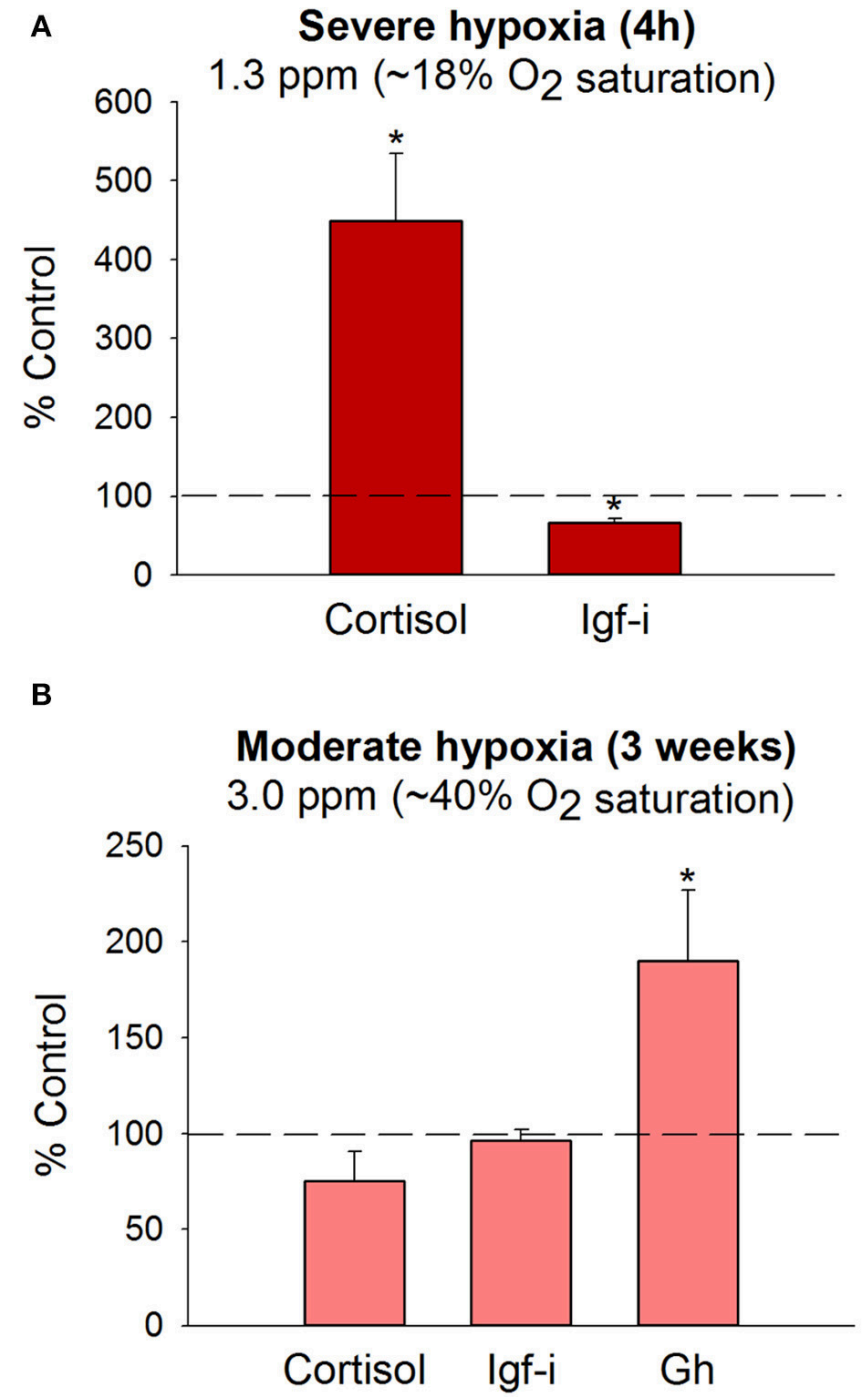
Plasma hormone levels in juveniles of gilthead sea bream following exposure to severe **(A)** or moderate hypoxia **(B)** at 20–22°C. Bars indicate changes in plasma cortisol, Gh and Igf-i levels in comparison to normoxic fishes (>85% O_2_ saturation). Data are presented as the mean ± SEM (*n* = 10–20). Asterisks indicate statistically significant differences (*P* < 0.05, *t*-test). Adapted from Martos-Sitcha et al. (134) and Magnoni et al. (140).

Fine adjustments of metabolism machinery also take place at the mitochondrial transcriptional level, and experimental evidence reveals a reduced production of energy and reactive oxygen species (ROS) when fishes are crowded (135) or exposed to multiple stressors that mimic daily aquaculture operations (136). In that sense, the capacity of fish to efficiently manage the allostatic load (defined as the maintenance of internal homeostasis through changes of a number of stress mediators) by readjustments of O_2_-carrying capacities and metabolic suppression are of high value to finally reach the internal equilibrium in a hypoxic-challenging scenario, which would prime a reduced production/accumulation of toxic byproducts from anaerobic metabolism (137–139). In hypoxic gilthead sea bream, this metabolic suppression is exemplified by the overall depression of catalytic, assembly, and regulatory enzyme subunits of complex I, II, III, and V of the mitochondrial respiratory chain, which is concurrent with the upregulation of catalytic and regulatory elements of complex IV (last electron donor to oxygen acceptor) (134). This dualism offers the possibility of a reduced but more efficient mitochondrial respiration during exposure or recovery from severe hypoxia, as has been shown, at least in part, in fishes fed with seaweed extracts as dietary surplus to protect against oxidative stress (140). Most of these metabolic readjustments, linked with endocrine disturbances (130, 134), have been reported after exposure to severe hypoxia (18–19% oxygen saturation, 20–22°C) for 4 h under steady-state conditions, but most analyzed parameters require lasting periods to be responsive when the water O_2_ concentration is fixed close to limiting oxygen saturation (LOS), defined as the threshold level where regulatory mechanisms are no longer sufficient to maintain the O_2_ consumption rate with changes of the level of O_2_ saturation (141, 142). Thus, juveniles of gilthead sea bream reared at 40% O_2_ saturation and 20–22°C exhibit a reduced, but active feeding behavior, that is adjusted to meet dietary O_2_ demands according to the oxystatic theory (143). This allows fishes to grow efficiently at slower rates, which is consistent with a total or partial recovery of control plasma levels of cortisol and Igf-i regardless of a persistent hypersomatotropic state that would reflect an enhanced demand for metabolic fuels (Figure 4). These endocrine signatures clearly indicate the different dynamics of the HPI and the Gh/Igf system in response to severe and moderate hypoxia, which merits consideration for metabolic phenotyping of farmed fishes in a scenario of increasing temperatures and global change.

### Nutritional background and Gh/Igf status

The current stagnation of fish meal (FM) and fish oil (FO) production from wild fisheries limits further growth of aquaculture (144). In the meantime, the most immediate alternatives are plant products, which have been used for years in salmonids and marine fish feeds to reduce the reliance of European aquaculture on marine fishery resources. Thus, most carnivorous farmed fish, including European sea bass (145) and gilthead sea bream (146, 147), can be successfully reared with plant-based diets containing < 10% marine feedstuffs. Moreover, complete replacement of FO by vegetable oils is feasible when the theoretical requirements for phospholipids and n-3 long-chain polyunsaturated fatty acids (LC-PUFA) are met by the lipids contained in FM (148–150), but the concomitant FM/FO replacement continues to be challenging despite the important research efforts within the framework of PEPPA (2001–2004), AQUAMAX (2006–2010), and ARRAINA (2012–2016) EU projects for meeting the nutrient requirements of European farmed fish in terms of growth, health, and welfare criteria. In that respect, important progress has been made in salmonid and non-salmonid fishes on the diagnosis of nutrient deficiencies or the re-evaluation of nutritional requirements of vitamins, minerals, and other key nutrients by means of surrogate markers resulting from blood biochemistry and hematology profiling, histopathological and organo-somatic index scoring or measures among others of enzyme activities or vertebral mineral concentrations (151–155).

Often, plant-based diets have an impact on the gastro-intestinal transcriptome, mucus intestinal proteome or intestinal microbiome, but the use of feed additives (e.g., butyrate) helps to preserve the wild phenotype of fish fed FM/FO-based diets, improving the disease outcomes of gilthead sea bream challenged with bacteria or intestinal parasites (156–158). Historically, the somatotropic axis has also been used as an endocrine marker of the effectiveness of alternative feed formulations to support maximum growth. A first study combining measures of circulating Gh, Igf-I, and Igfbps with those of Gh-binding and *gh* and *igf* transcripts was conducted by Gómez-Requeni et al. (78) to investigate the physiological consequences of the partial or total replacement of FM by plant ingredients in trout juveniles. Likewise, measurements of circulating Gh and Igf-i in combination with those of transcripts of *ghrs, igfs, igfbps*, and other growth-related markers, including molecular chaperones, myogenic factors, energy sensors, and markers of protein turnover, lipid metabolism, oxidative phosphorylation (OXPHOS) and mitochondria respiration uncoupling, are highly informative for a wide-ranging assessment of growth performance; first with the replacement of FM by plant proteins (159) and second with the combined and maximized replacement of FM and FO by plant proteins and vegetable oils (146, 160–162). This results in differences in growth rates with a high degree of concordance of growth and growth-promoting factors between trials (9–11 weeks) conducted during the summer growing period with juvenile fishes of the same strain (Atlantic fish strain) and class of size (14–16 g initial body weight).

**Figure 5:**
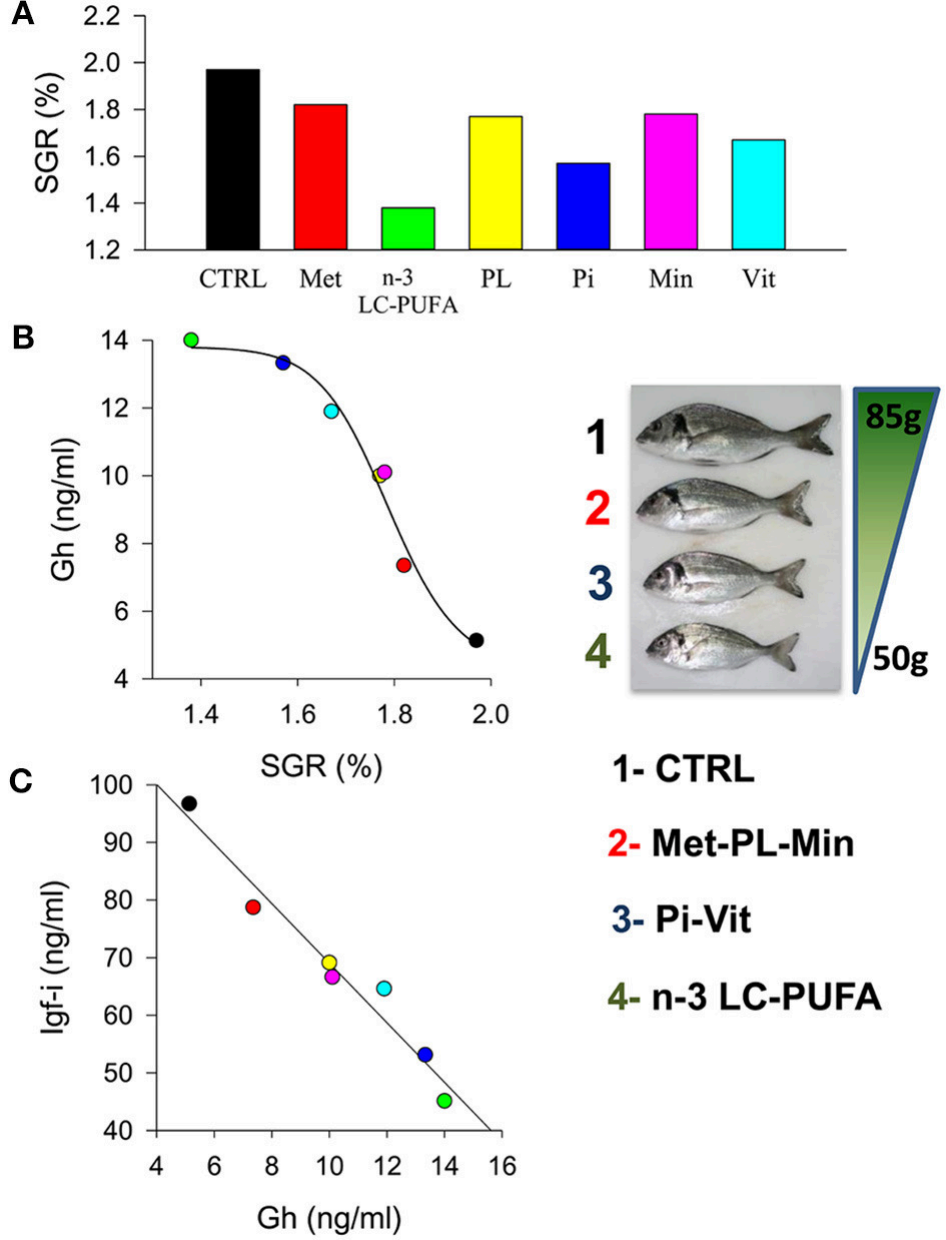
**(A)** Effect of nutrient deficiencies on the specific growth rates (SGR) in juveniles of gilthead sea bream fed to visual satiety for 13 weeks from May to July. **(B)** Curvilinear correlation between SGR and plasma Gh levels. Insert image shows fish size differences at the end of trial. **(C)** Correlation between plasma Gh and Igf-i levels. Colors indicate each experimental group: CTRL, control (black); Met, methionine (red); n-3 LC-PUFA, n-3 long-chain polyunsaturated fatty acids (green); PL, phospholipids (yellow); Pi, phosphorus (blue); Min, minerals (pink); Vit, vitamins (cyan). Adapted from Ballester-Lozano et al. (152).

The above observations further support a consistent growth output for a given Igf-i concentration that makes feasible meta-analysis of data from highly controlled experiments. In that sense, the evolutionary past of living animals is marked by periods of undernutrition that are characterized by protein-calorie deficiencies or vitamin and mineral deficiencies and even starvation, which makes survival dependent on the body's ability to mobilize energy stores (163). Since GH plays a key role in mobilizing energy (lipolytic role non-dependent of IGFs) its elevated circulating levels in the setting of low IGF-I confers a metabolic advantage during undernutrition periods. Therefore, a state of GH resistance is highly conserved through the evolution of fishes and higher vertebrates as part of the adaptive response to inappropriate nutritional conditions. This reflects a reduced responsiveness of target tissues to the anabolic GH action, which may be due to receptor or postreceptor defects in the transmission of GH signaling. Thus, diets deficient in tryptophan resulted in reduced growth and plasma Igf-i in juveniles of the hybrid striped bass (164). Hevrøy et al. (165) also found that lysine-enriched diets resulted in a significant increase in muscle protein deposition and hepatic *igf-i* mRNA in Atlantic salmon. Likewise, methionine availability modulates the expression of genes involved in the Gh/Igf response and protein turnover, further affecting growth performance in trout (166). Previous studies in trout indicate that plasma Gh level is not affected by either methionine or taurine supplementation, and even more, methionine excess has been associated with decreased plasma Igf-i in fishes fed FM-free diets (167). This apparent discordance indicates the complexity of endocrine growth regulation within and between fish species. However, it is noteworthy that recent studies in gilthead sea bream highlight a close linear relationship between growth and circulating levels of Gh and Igf-i in fishes fed semipurified diets formulated for deficiencies in methionine, n-3 LC-PUFA, phospholipids, phosphorus, vitamins, or minerals (Figure 5). This association between growth and Gh/Igf-i is also concordant with that found in juveniles fed practical diets with varying degrees of FM and FO replacement. Therefore, the somatotropic axis highly reflects the drawback effects on growth performance due to a reduced nutritive value of the main dietary protein or lipid sources, but also in response to specific nutrient deficiencies mimicking those induced by enriched plant-based diets.

More recently, it was found that the growth-promoting action of medium-chain fatty acids in juveniles of gilthead sea bream fed FO-based diets resulted in a slight increase in the Igf-i/Gh ratio (168). In this way, the combined use of circulating Igf-i and the Igf-i/Gh quotient have been revitalized as reliable key indicators of growth performance in fishes, highly reflecting the magnitude of a wide range of nutritional and growth derangements. Certainly, circulating Gh and Igf-i are either the up- or downstream factors of the Gh/Igf system, but they are informative of the intensity of growth impairment rather than the nature or origin of disturbing processes. Hence, as indicated below, measures of transcripts of the main actors of the somatotropic axis alone or in combination with other markers of protein mass accretion and cellular stress are required for a refined diagnosis of a given nutrient deficiency or even for the re-evaluation of species-specific nutrient requirements.

## Regulation at the transcriptional level

### Effects of nutrition and season on *ghrs*

Fish *ghrs* were first cloned and sequenced in goldfish (169), turbot (170), and sparid fishes (171, 172). Nucleotide sequences encoding for fish *ghrs* were also available in coho and masu salmon at the end of the last century, and several authors suggested a divergent evolution of salmonid and non-salmonid *ghr*s. However, as first pointed out by Saera-Vila et al. (173), duplicated fish *ghrs* are actively transcribed in trout, European sea bass or gilthead sea bream and they are more similar to each other than to *Ghr*s of tetrapods. Searches for *ghrs* in fish genome databases also confirmed their occurrence in almost all the analyzed fishes, but unfortunately the initial controversy over *ghr* phylogeny has made the nomenclature of fish *ghrs* somewhat confusing. To solve this issue, the convention for fish *ghr* nomenclature is the clade *ghr* type I (also written type 1 or *slr* in masu salmon) for the initially described *ghr* of non-salmonid fishes, while the clade *ghr* type II (also written type 2) corresponds to the initially described *ghr* isotype of salmonids [see Reindl and Sheridan (60)], where the chromosomal location of duplicated sequences of *ghrs* of type I and II are consistent with the generation of paralogous blocks in the salmonid tetraploidization (174).

**Figure 6:**
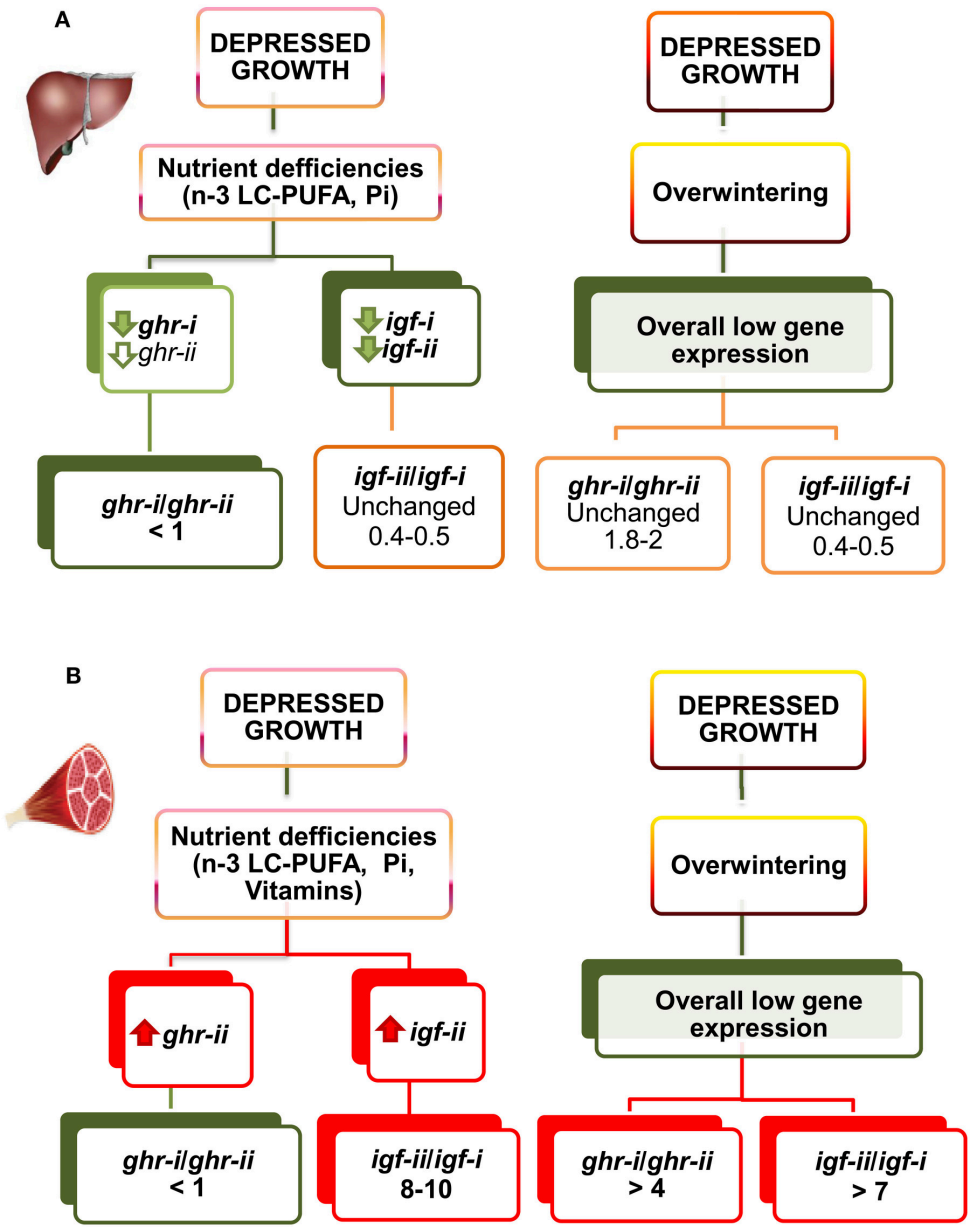
Regulated gene expression of *igfs* and *ghrs* by diet composition and season in liver **(A)** and skeletal muscle **(B)** of gilthead sea bream juveniles. The direction of change is represented by color (red, increase; green, decrease; orange, unchanged). For full details of gene expression profiling see Tables S1–S6. Adapted from Benedito-Palos et al. (146, 160) and Ballester-Lozano et al. (152).

Most of what we know about GH signaling comes from mammalian species and cell lines, but intracellular signaling pathways are generally well conserved, and many commercially-available antibodies developed to target mammalian signaling molecules also detect orthologs in piscine species (175). This opens new research opportunities, and several attempts have been made to support the possibility that a given Ghr subtype might be responsible for transmitting the lipolytic action of Gh, while the other Ghr subtype would be more active in transmitting the growth-promoting action of Gh (31). To our knowledge, more work is needed to establish these explicit links, though it is well-recognized that Gh differentially activates disparate signaling pathways when stimulating growth through Jak-sat, Pi3k-akt, and Mapk (176), and lipolysis through Plc-pkc, MapK, and Hsl (177–179). Meanwhile, the differential and tissue-specific regulation of *ghr* subtypes by nutritional and environmental factors becomes especially clear in farmed gilthead sea bream, in which hepatic transcripts of *ghr-i* (in a low extend *ghr-ii*) mirror changes in growth rates, plasma Igf-i level, and hepatic *igf-i* transcription through development and production cycles, indicating a prominent role of Ghr-i rather than Ghr-ii in the systemic growth-promoting action of Gh (73, 146, 160). Moreover, the hepatic *ghr-i/ghr-ii* gene expression ratio remains mostly unaltered during seasonal changes of growth rates. However, this gene expression ratio is upregulated in white skeletal muscle from 2 to 3 in summer to more than 4 in winter. This gene expression ratio is also highly regulated at the nutritional level, and fishes fed unbalanced plant-based diets or semipurified diets formulated for specific nutrient deficiencies downregulate hepatic *ghr-i*, decreasing the *ghr-i/ghr-ii* ratio from 1.8 to 2 to < 1 (146, 152, 160). Likewise, in white skeletal muscle, the *ghr-i/ghr-ii* ratio decreases from 2.5 to 3 to close to 1 in fishes with signs of nutrient deficiencies, but this molecular feature seems to be the result of the counterregulatory upregulated expression of *ghr-ii*. Conversely, impaired growth during overwintering is not able to alter significantly the hepatic *ghr-i*/*ghr-ii* mRNA ratio regardless of the overall depressed gene expression during the cold season (see Figure 6).

### Insulin/Igf system: evolutionary prospect

**Figure 7:**
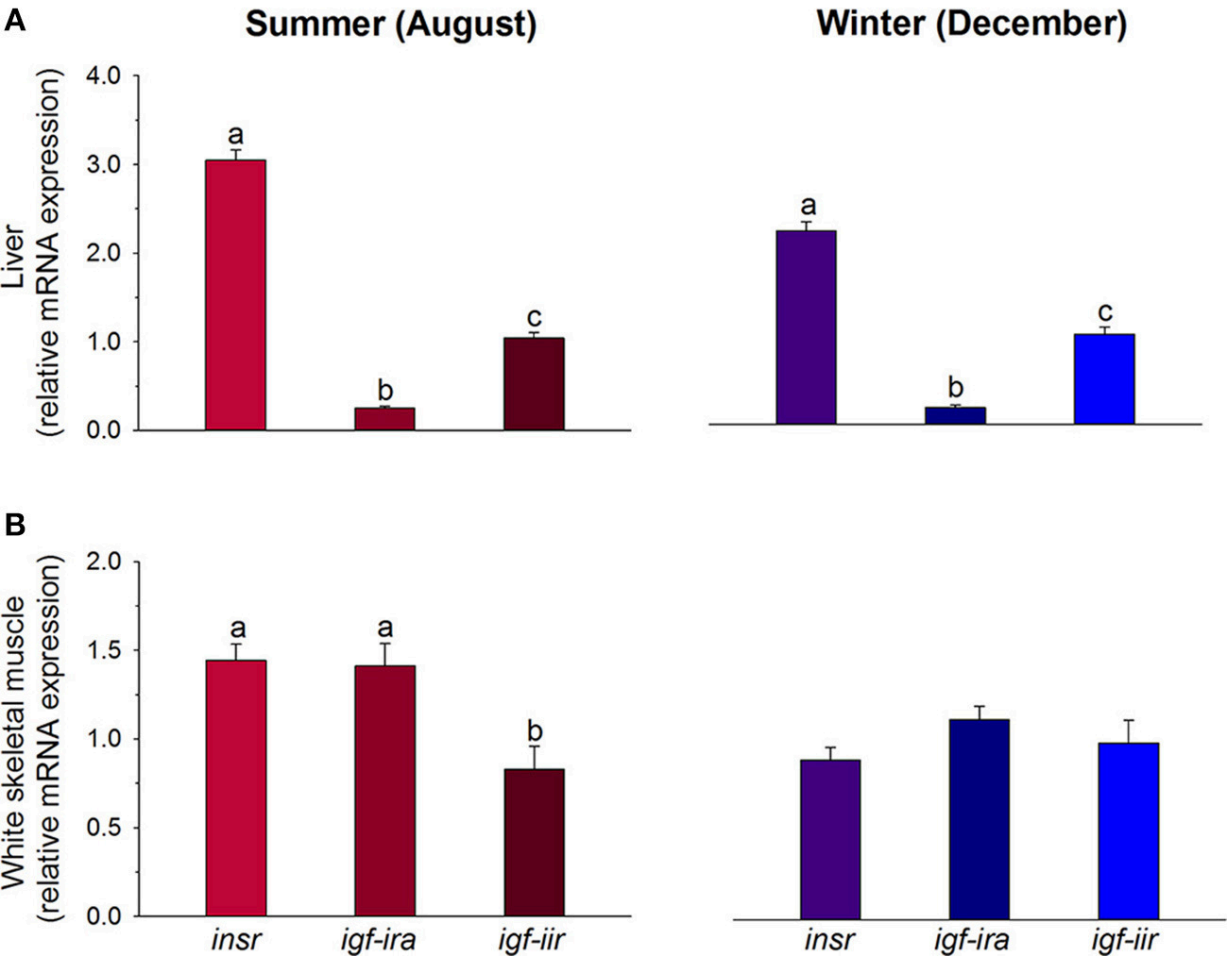
Regulated gene expression of receptors of insulin (*insr*), *igf-i* (*igf-ira*), and *igf-ii* (*igf-iir*) in liver **(A)** and skeletal muscle **(B)** of gilthead sea bream juveniles during summer (red colors) and winter (blue colors). For each tissue and season, data values (mean ± SEM, *n* = 6–7) are normalized to the expression level of *igf-iir* (arbitrary value of 1). Different superscript letters indicate significant differences (*P* < 0.05; ANOVA followed by Student-Newman–Keuls test). Data derived from samples from Benedito-Palos et al. (146).

Insulin, IGF-I and IGF-II are structurally similar and they are derived from a common ancestral molecule through a series of gene duplications and mutations (180). Fishes are the first group in the vertebrate tree in which there is evidence of distinct insulin and Igf molecules and receptors (181). However, certain cross-interaction between ligands and receptors of insulin and Igfs occurs. This is especially evident for Igf-ii, which exerts its mitogenic action through insulin and Igf-i receptors (182, 183). Additionally, important differences regarding receptors specificity and abundance through the evolution have been reported. Indeed, fish Igf-i receptors show a higher degree of specificity than insulin receptors (184, 185), and binding studies in cardiac and skeletal muscle highly support that the number of insulin receptors is lower than the number of Igf-i receptors in fishes, amphibians and reptiles (186–188), whereas the opposite is found in birds and mammals (189, 190). Nevertheless, at the expression level, the relative abundance of insulin receptors in juveniles of gilthead sea bream fed practical diets from early life stages is higher than initially expected. This is especially evident in liver, where the expression quotient ratio for insulin receptors and Igf receptors remains almost equal along seasons. The same trend, but less evident, is found in white skeletal muscle, where the expression level of insulin receptors is almost equal to that of the *igf-i* receptor in both summer and winter, and higher than that of the *igf-ii* receptor in summer (see Figure 7). Since gilthead sea bream is an euryhaline, eurythermal, and protandrous hermaphrodite fish, it is tempting to speculate that the increase in the insulin receptor/*Igf* receptor expression ratio with organismal complexity from ectotherms to endotherms also applies to a fish with a well-recognized growth and metabolic plasticity, and improved resilience to aquaculture stressors (112, 135, 136, 191).

### *igf-ii/igf-i* expression ratio

A growing body of evidence from more than 25 years indicates that IGFs play key roles in the growth and development of mammals and chickens. As a result of multiple transcription initiation sites and alternative splicing, the *Igf-i* gene gives rise to different transcripts in higher vertebrates (192, 193), encoding for several Igf-i precursor polypeptides. The biological significance of these splice variants still remains under debate, although a differential expression profile has been reported in response to varying conditions and pathologies (194, 195) and potentially different bioactivity of the Igf-i isoforms is suggested. Multiple forms of Igf-i have been also detected in fishes, including gilthead sea bream (196–199). This functional plasticity is not found in IGF-II, which is considered a primary growth factor for embryonic and fetal growth (200, 201), while IGF-I is required for achievement of maximal postnatal growth (202). Indeed, postnatally elevated levels of *Igf-ii* transcripts fail to rescue the dwarfism of *Igf-i*-deficient mice (203), and *Igf-ii* transcripts decrease quickly during the postnatal development of mice and rats (204). However, substantial *IGF-II* amounts are found later in life in humans and in a wide-range of fishes, including common carp (205), trout (206), Nile tilapia (207), channel catfish (208), and gilthead sea bream (209). Overexpression of *igf-ii* is especially evident in extrahepatic tissues: in gilthead sea bream, the *igf-ii/igf-i* expression ratio ranges from 0.5 in liver and 3–9 in skeletal muscle, adipose tissue, gills and brain to 75–150 in heart, intestine and gonads. In these studies, *igf-i* measures considered the expression of the totality of transcripts given the retention of the core mature peptide in all Igf-i precursors (159).

Regarding in depth *igf-ii* expression, compensatory increases in skeletal muscle *igf-ii* mRNA also occurs in juveniles of gilthead sea bream fed FO-free diets to counteract, at least in part, the suppressed growth and expression of hepatic *igfs* (160). However, more recent data using fishes fed semipurified diets formulated for nutrient deficiencies indicate that this type of muscle response is perhaps more informative of deficiencies in vitamins rather than n-3 LC-PUFA. The *igf-ii/igf-i* expression ratio is also sensitive to seasonal changes in growth rates, and the apparent winter suppression of muscle *igf* expression is more evident for the lowest-expressed *igf* gene. The *igf-ii/igf-i* ratio in juvenile fishes varies from 10 in summer to 3–5 in winter. By contrast, the hepatic *igf-ii/igf-i* ratio remains almost unaltered and near 0.5 over season in both well and malnourished fishes. This is because the two hepatic *igf* transcripts are similarly regulated when facing different environmental and nutritional stimuli (see Figure 6). The regulation of *igf* expression quotient is, therefore, different from that reported for *ghrs*, as the expression of *ghr-i* is more regulated in liver than in skeletal muscle, whereas *ghr-ii* and *igf-ii* appear more regulated at the muscle local level, as part of some kind of compensatory growth response. This finding helps to clarify the different functions of *ghr* and *igf* duplicated genes, helping to refine the molecular signatures of a given growth and nutritional condition through production cycles.

### Fish *igfbp* repertoire

**Figure 8:**
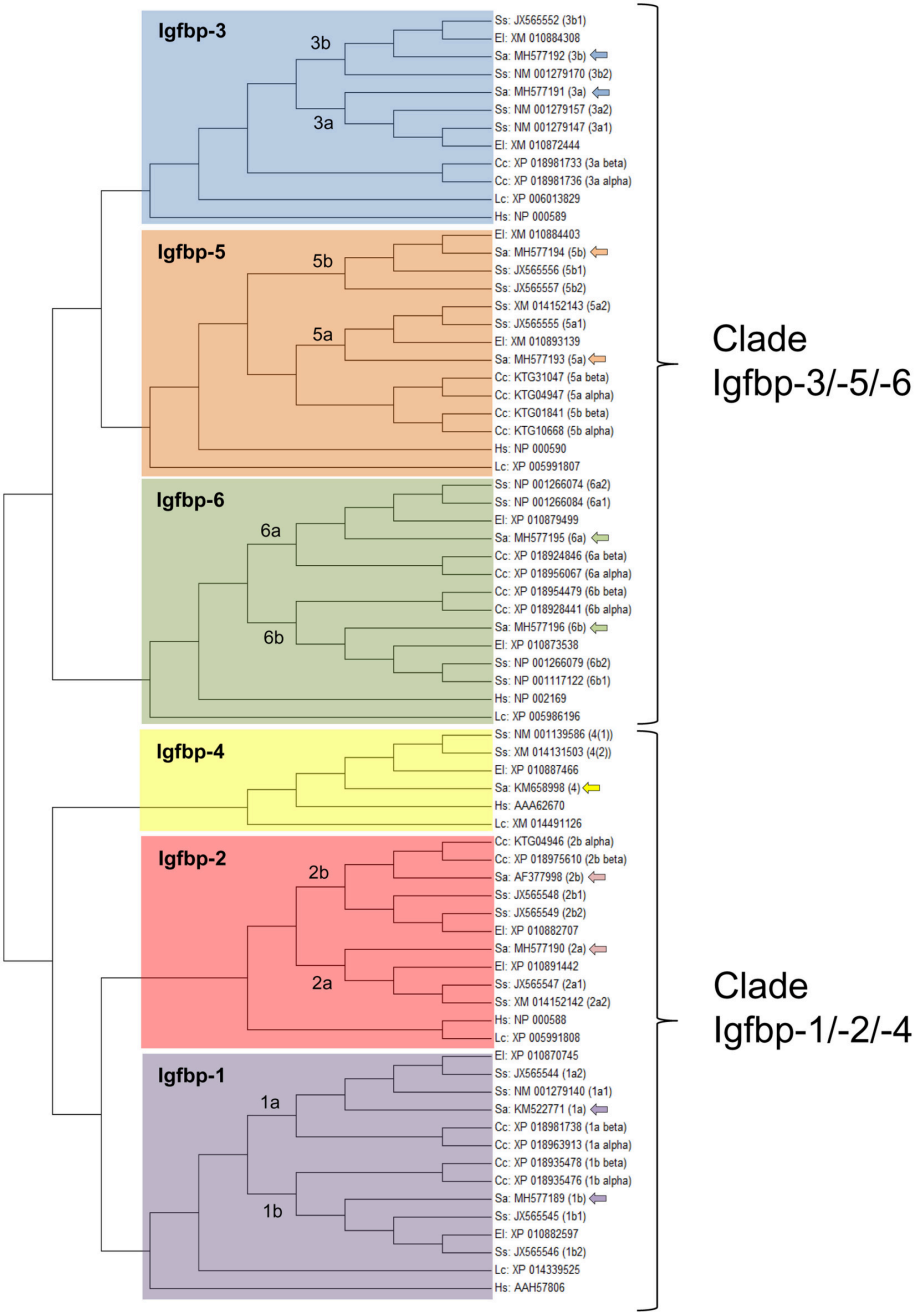
Phylogenetic analysis of the Igfbp family, evidencing the two Igfbp modern vertebrate clades and the expansion of the family in teleosts. The unrooted tree was constructed by means of Mega 6.0 using the maximum likelihood method, with the amino acid alignment of 72 Igfbp sequences from Human, *Homo sapiens* (Hs); Comoran coelacanth, *Latimeria chalumnae* (Lc); northern pike, *Esox lucius* (El); carp, *Cyprinus carpio* (Cc); Atlantic salmon, *Salmo salar* (Ss); and gilthead sea bream, *Sparus aurata* (Sa). GenBank accession numbers are given for all sequences. Duplication events in teleosts are shown. The 11 gilthead sea bream sequences, including *igfbp-4* and paralog pairs of *igfbp-1, -2, -3, -5*, and *-6* are marked with arrows.

The ancestral *Igfbp* gene was duplicated in tandem during an early stage of vertebrate evolution to produce a pair of *Igfbp*s that gave rise in subsequent genome duplication events the two *Igfbp* clades of modern vertebrates (*Igfbp-1/-2/-4*; *Igfbp-3/-5/-6*) (210–213). Additionally, the third and fourth round of whole-genome duplications created the corresponding paralog pairs. The resulting number of Igfbp subtypes is thus variable between fish lineages, but always higher than in mammals and other vertebrates. For example, zebrafish have retained nine actively transcribed genes that include *igfbp-3a* and paralog pairs of *igfbp-1, -2, -5*, and *-6* (214–217). Atlantic salmon possesses 22 unique *igfbp* genes with 11 paralog pairs of *igfbp-1a, -1b, -2a, -2b, -3a, -3b, -5a, -5b, -6a*, and *-6b*. Common carp retains 17 *igfbp* genes including *igfbp-2a* and paralog pairs of *igfbp-1a, -1b, -2b, -3a, -5a, -5b, -6a*, and *-6b* (218). Likewise, searches in the gilthead sea bream genome database (http://nutrigroup-iats.org/seabreamdb/) have identified 11 *igfbp* genes, covering the full *igfbp-1* to *-6* repertoire with paralog pairs of *igfbp-1, -2, -3, -5*, and *-6*. These findings evidence a different and perhaps divergent evolution of the *igfbp* repertoire in fish species, though high quality reference genomes, with all genes properly annotated and assembled into corresponding chromosomes, are required to confirm this idea. At this stage, to avoid nomenclature confusion, new and previous *igfbp* sequences of gilthead sea bream are annotated, and uploaded to GenBank, according to the proposed nomenclature of salmonids. The identity of the annotated *igfbp* sequences has been corroborated by phylogenetic analyses, which evidenced the differential expansion of *igfbp* genes within salmonids, carp, northern pike and gilthead sea bream (Figure 8).

**Figure 9:**
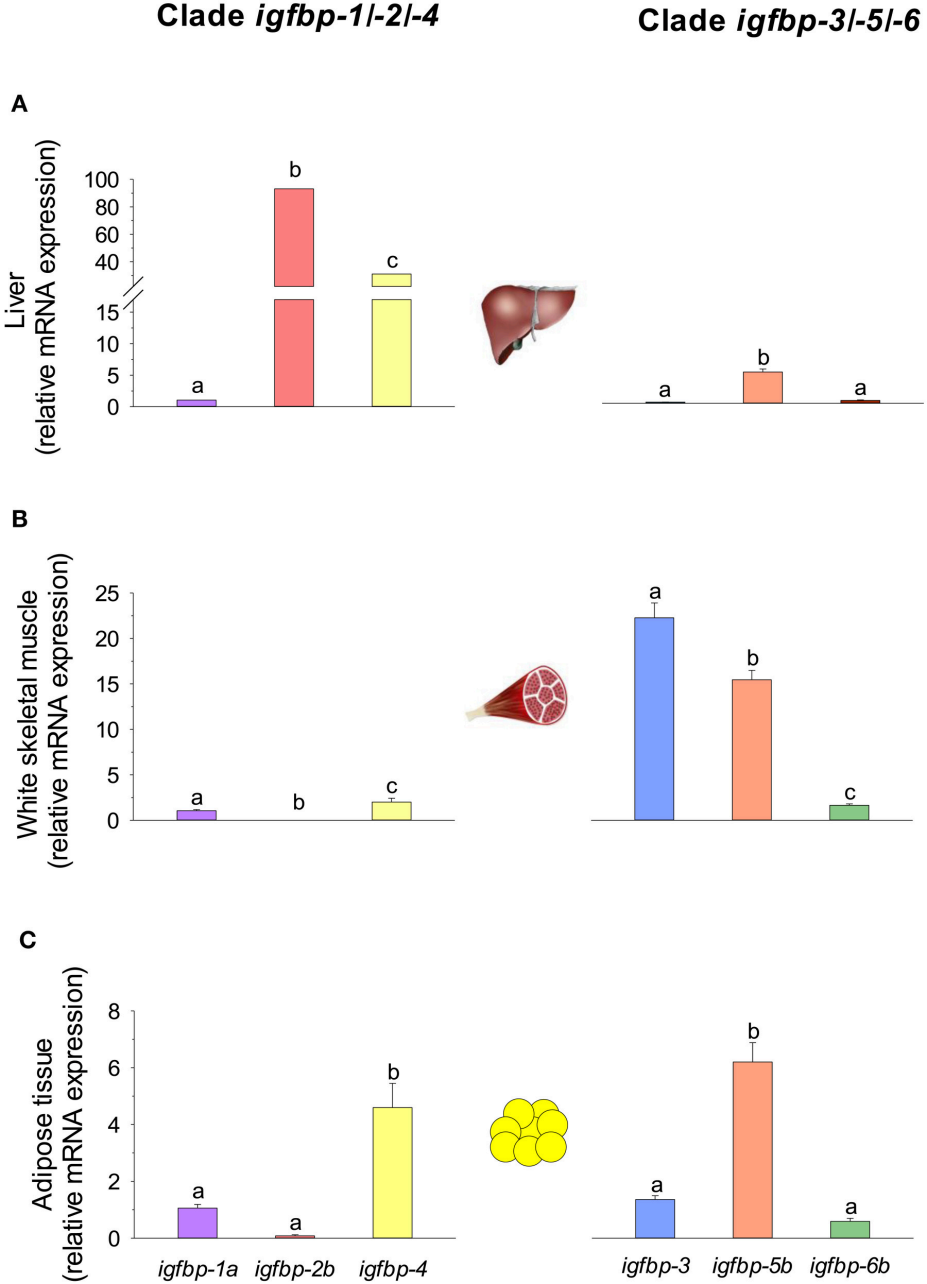
*igfbp* gene expression profile in **(A)** liver, **(B)** white skeletal muscle, and **(C)** adipose tissue of juveniles of gilthead sea bream. RT-qPCR of tissue total RNA was conducted as previously reported (134). Analyzed genes of clade *igfbp-1/-2/-4* were *igfbp-1a, igfbp-2b*, and *igfbp-4*. For clade *igfbp-3/-5/-6*, primers for *igfbp-3* detected both *igfbp-3a* and *-3b* paralogs, and expression levels of *igfbp-5b* and *igfbp-6b* were analyzed. For each tissue, data values (mean ± SEM, *n* = 8–9) are normalized to the expression level of *igfbp-1a* (arbitrary value of 1). In each tissue and clade, different superscript letters indicate significant differences (*P* < 0.05; ANOVA followed by Student-Newman–Keuls test).

Regarding the expression profile, the clearest pattern is the different tissue-specific profile of *igfbps* in liver and skeletal muscle. Of note, transcripts of the *igfbp-1/-2/-4* clade are highly represented in the liver tissue of gilthead sea bream juveniles: *igfbp-2b* and *igfbp-4* comprise more than 70 and 20% of *igfbp* mRNAs, respectively. In contrast, the *igfbp-3/-5/-6* clade is overrepresented in skeletal muscle, and *igfbp-3* and *igfbp-4* transcripts make up 90% of mRNAs coming from *igfbp* genes. This clear divergence of expression patterns for the two *igfbp* clades is also a characteristic feature of Atlantic salmon in liver and muscle tissues (213). By contrast, this dichotomy is not retained by the adipose tissue where a relatively high expression level is found for both *igfbp-4* (*igfbp-1/-2/-4* clade) and *igfbp-5b* (*igfbp-3/-5/-6* clade) genes in gilthead sea bream (Figure 9). In blood, the presence of different Igfbps within a molecular range of 20–50 kDa has been reported by Igf-binding assays in various fish species but, unlike in mammals, Igfbp-2 and not Igfbp-3 is emerging as the major blood Igf-i carrier in fish species (219), which is consistent with the elevated expression pattern of *igfbp-2* genes in the liver tissue of this group of vertebrates.

**Figure 10:**
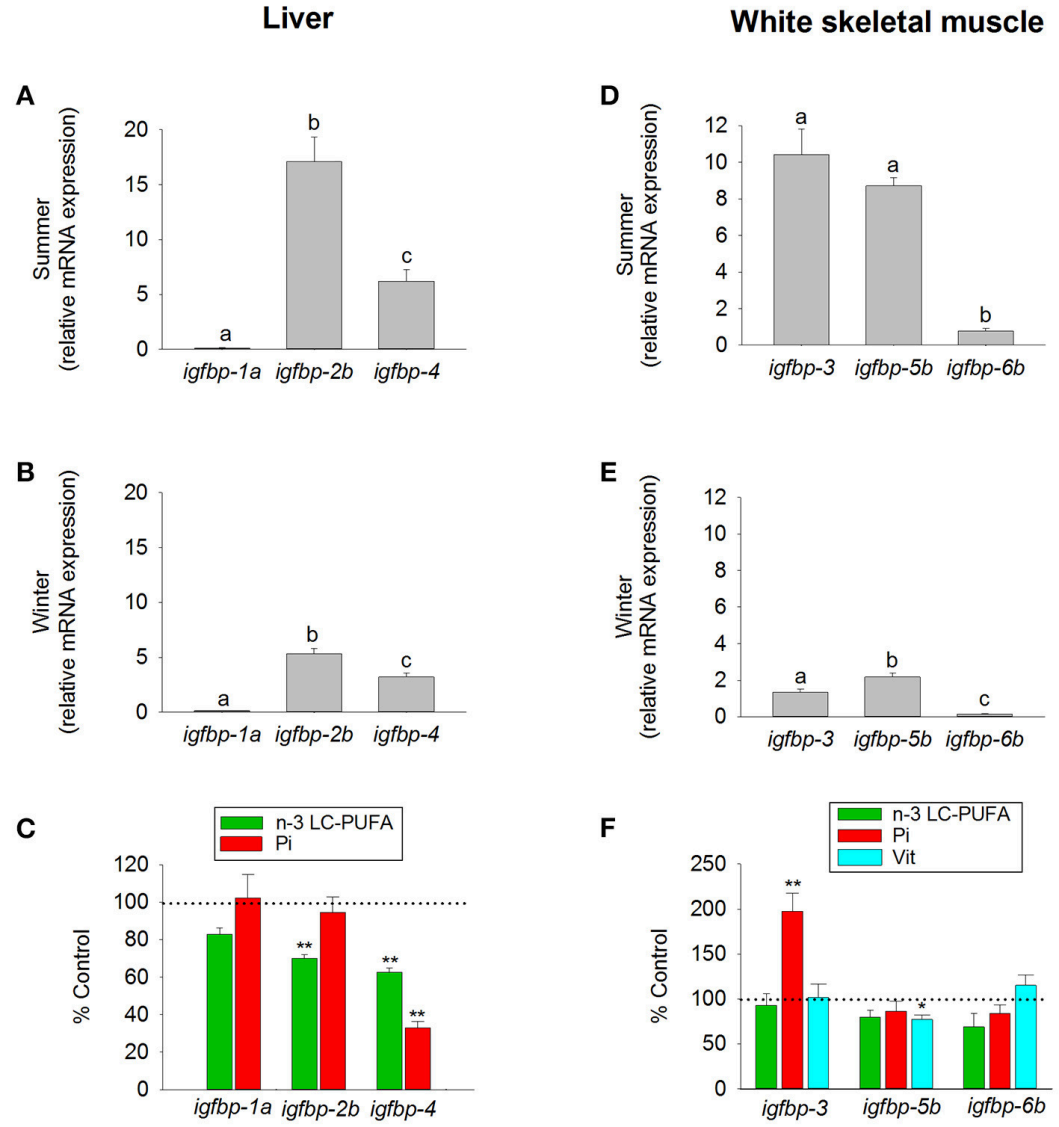
Tissue-specific regulation of *igfbp* by season and diet in juveniles of gilthead sea bream. Liver expression of *igfbp-1a, igfbp-2b*, and *igfbp-4* (clade *igfbp-1/-2/-4*) was analyzed in fishes during **(A)** summer, **(B)** winter, and **(C)** after a dietary challenge with a diet deficient in n-3 LC-PUFA (green bars) or phosphorous (Pi, red bars). White skeletal muscle expression of *igfbp-3* (primers detected both *igfbp-3a* and *-3b* paralogs), *igfbp-5b*, and *igfbp-6b* (clade *igfbp-3/-5/-6*) was analyzed in fishes during **(D)** summer, **(E)** winter, and **(F)** after a dietary challenge with a diet deficient in n-3 LC-PUFA (green bars), phosphorus (Pi, red bars) or vitamins (Vit, cyan bars). RT-qPCR of tissue total RNA was conducted as previously reported (134). Data are represented as the mean ± SEM (*n* = 6–7). For each tissue and season **(A,B,D,E)**, different superscript letters indicate significant differences (*P* < 0.05; ANOVA followed by Student-Newman–Keuls test). Asterisks in **(C,F)** indicate significant differences compared with paralog expression in fishes from group fed a control diet (*t*-test, *P < 0.05; **P < 0.001). Data derived from samples from Benedito-Palos et al. (146) and Ballester-Lozano et al. (152).

More controversial are the main roles of each IGFBP as a growth-promoting or inhibiting factor through their IGF- or non-IGF-mediated effects. Recently, this has been extensively reviewed (210, 218), though it remains difficult to draw a general conclusion since most of the physiological roles, when conserved, differ across species and physiological contexts. Moreover, the lack of substantial phenotypes when *Igfbp*-*3*, -*4*, and -*5* were knocked out together in mice suggests a high degree of functional redundancy and/or genetic compensatory mechanisms (220), which reflects versatile modes of regulation in response to specific stressful or aberrant conditions. In any case, a large body of evidence in higher vertebrates and fasted, refed, or *gh*-transgenic fishes supports a main role of Igfbp-1 as a negative regulator of teleost growth. For instance, knockdown of *igfbp-1* alleviates the hypoxia-induced growth retardation and development delay in zebrafish, whereas overexpression of *igfbp-1* causes growth and developmental retardation under normoxia (221). Along the same lines, we found that hepatic *igfbp-1a* expression remains mostly repressed in grow-out gilthead sea bream during both summer and winter periods (Figures 10A,B). Mixed results exist on the regulation of *Igfbp-2* genes. Mouse embryos overexpressing *Igfbp*-*2* show a reduced growth rate, likely through reduced IGF availability (222). By contrast, work in Atlantic salmon highlights an increase in circulating Igfbp-2 in response to Gh (218), while early studies in zebrafish reported that Gh inhibits *igfbp-2* expression (223). As in salmonids, data on *igfbp-2b* expression in gilthead sea bream support a growth-promoting action of Igfbp-2, which is substantiated by its seasonal expression pattern and its depressed expression in fishes with signs of essential fatty acid deficiencies (Figure 10C). On a seasonal basis, the expression pattern of *igfbp-4*, recognized as a growth-promoting factor in salmonids (218), is similar to that of *igfbp-2b* at a lower expression level, but importantly the regulation of *igfbp-4* appears especially sensitive to nutrient deficiencies in essential fatty acids and phosphorus (Figures 10A–C).

Igfbp-3 seems to have less of a common role among fish lineages. Indeed, studies in zebrafish, flounder and yellowtail reported a significant increase in the expression level of *igfbp-3* in liver and/or muscle in response to fasting (224–226), which may act to restrict Igf signaling. By contrast, studies in trout and Atlantic salmon have reported no changes in the expression of *igfbp-3* in response to fasting (63, 218), whereas the muscle expression of *igfbp-3a1* is enhanced by *gh*-transgenesis in coho salmon (227), which is consistent with a growth-promoting action in salmonids. To make matters more complicated, in gilthead sea bream, the expression of muscle *igfbp-3* is elevated not only during the summer growth enhancement but also during growth impairments due to phosphorus deficiencies, which supports both growth-promoting and -inhibiting roles depending on the physiological context. In contrast, the main role of Igfbp-5b is to promote growth in response to changes in gene expression with the season and nutritional status (Figures 10D–F). Overall, the available evidence for Igfbp-5 supports a growth-promoting function in salmonids, but again functional divergence has been reported across species and physiological contexts [see García de la Serrana and Macqueen (218)]. Conversely, Igfbp-6 is emerging as a growth-inhibitory factor, though this Igfbp is particularly understudied, and it is difficult to draw overarching conclusions. However, taking into account its relatively low expression in the liver and skeletal muscle of well-nourished fishes, the proposed role for Igbp-6b in gilthead sea bream is closer to a growth-inhibiting rather than a growth-promoting action.

## Concluding remarks and prospects

The GH/IGF system plays a key role in the endocrine cascade of growth, and overall changes at the protein and mRNA levels closely reflect differences in growth performance through development and production cycles. However, this relationship varies within and across fish species and physiological contexts from tight to non-discernible correlations due to the actions of a wide range of endogenous and exogenous factors. Despite this, changes in circulating GH and IGF-I levels continue to be one of the most robust markers of growth performance through vertebrate evolution. This is especially relevant in fish species since gene expansion by local or whole-genome duplications offers the possibility of a complex gene subfunctionalization and/or acquisition of novel functions. This is evidenced at the ligand level for Gh and Sl and at the receptor level for the *ghr-i* and *ghr-ii* genes, which are differentially regulated in liver and skeletal muscle, helping to distinguish stressful and growth disturbances due to overwintering or malnutrition as a result of changes in feed intake, protein and lipid feedstuffs or any other specific nutrient. Close links between oxygen availability, energy status and the somatotropic axis are also now emerging via Sirts, which are potential markers of informing of energy status and can modulate the anabolic action of Gh by inhibiting Ghr signaling. The differential regulation of *igf-i* and *igf-ii* genes in liver and skeletal muscle also offers the possibility of a more refined analysis of growth potentiality and nutritional condition. Igfbps are emerging as highly regulated components of the Gh/Igf system, though the puzzle is far from complete because their tissue-specific regulation has not been established for all paralogs across fish species and different physiological conditions. Thus, further research is needed to combine the search for a robust, highly specific set of biomarkers for a given growth derangement, which may have an impact later in life by means of different epigenetic mechanisms, involving changes in DNA methylation and histone acetylation, among others (228).

**Figure 11:**
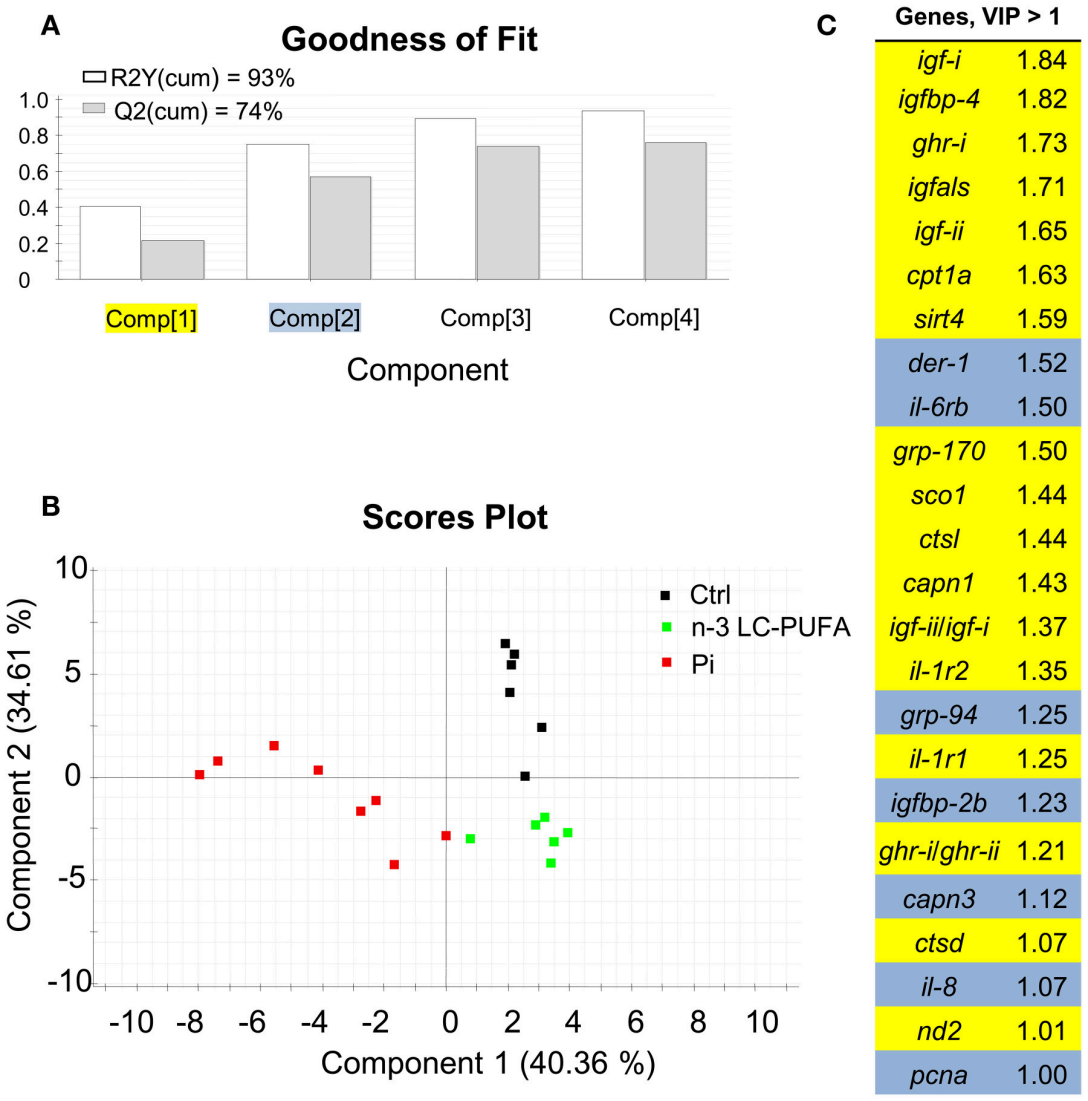
Discriminant analysis (PLS-DA) of liver molecular signatures of fishes fed nutrient deficient diets. Relative expression data of the 73 genes included in the array can be found in Table S1. **(A)** Cumulative coefficients of goodness of fit (*R*^2^, white bars) and prediction (*Q*^2^, gray bars) by each component; the two first components explained 74.97% of total variance. **(B)** PLS-DA score plot of acquired data from dietary challenged individuals along the two main components. Individuals fed the phosphorus deficient diet (Pi, red squares) are separated along the first component, and component 2 separates individuals fed the control (Ctrl, black squares) and LC-PUFA-deficient diets (green squares). **(C)** Ordered list of markers by variable importance (VIP) in the projection of PLS-DA model for group differentiation. Markers with VIP values >1 after the first or second component are highlighted in yellow and blue, respectively. Data derived from samples from Ballester-Lozano et al. (152).

**Figure 12:**
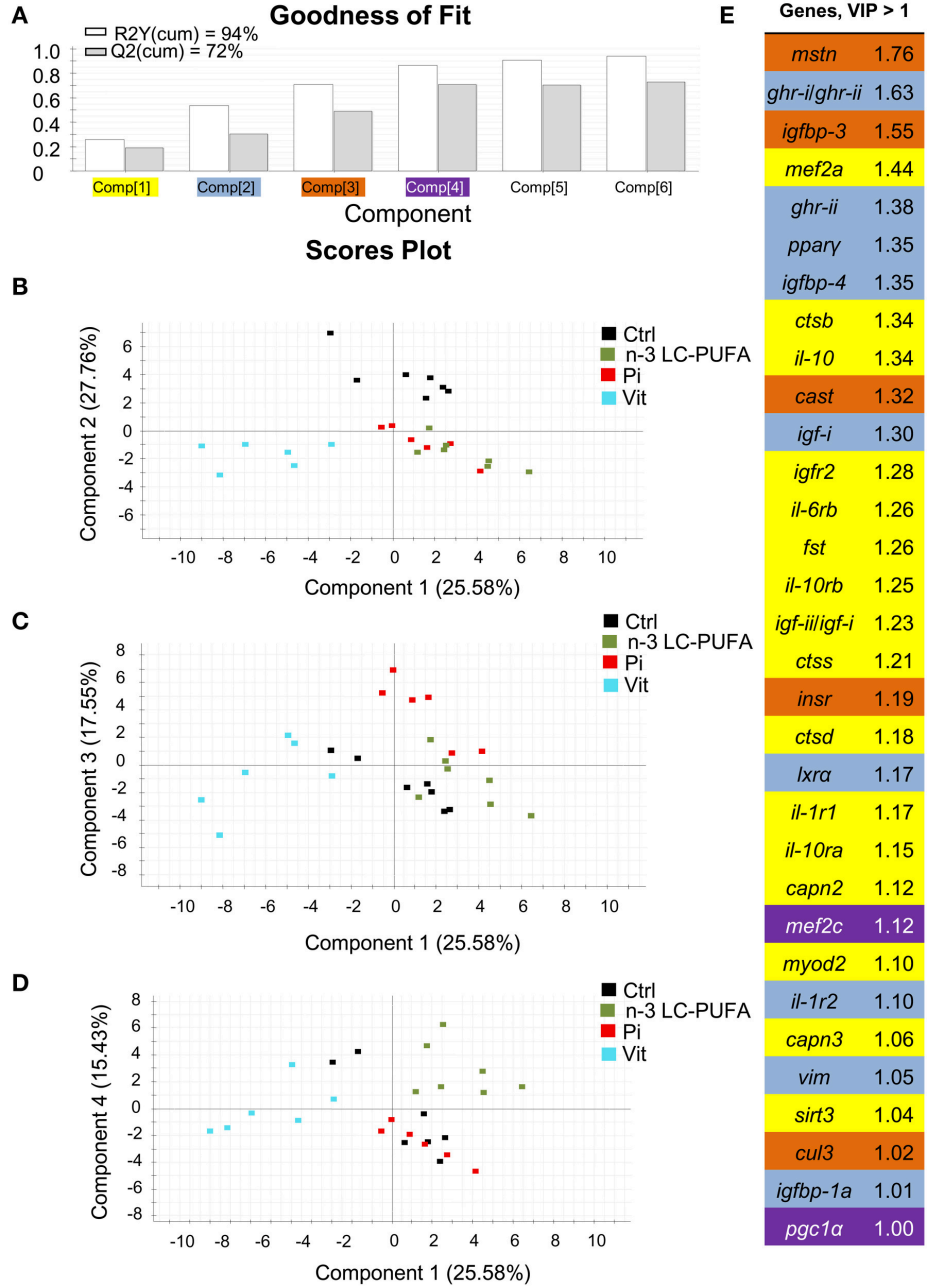
Discriminant analysis (PLS-DA) of skeletal muscle molecular signatures of fishes fed nutrient deficient diets. Relative expression data of 84 genes can be found in Table S2. **(A)** Cumulative coefficients of goodness of fit (*R*^2^, white bars) and prediction (*Q*^2^, gray bars) by each component; 86.32% of total variance is explained by four components. **(B)** PLS-DA score plot of acquired data from dietary-challenged individuals along components 1 and 2. Individuals fed the vitamin-deficient diet (Vit, cyan squares) are separated along the first component. **(C)** PLS-DA score plot of acquired data from dietary-challenged individuals along components 1 and 3. Component 3 separates individuals fed the phosphorus-deficient diet (Pi, red squares). **(D)** PLS-DA score plot of acquired data from dietary-challenged individuals along components 1 and 4. Component 4 separates individuals fed the LC-PUFA-deficient diet (green squares). **(E)** Ordered list of markers by variable importance (VIP) in the projection of PLS-DA model for group differentiation. Markers with VIP values >1 after the first, second, third and fourth components are highlighted in yellow, blue, orange and purple, respectively. Data derived from samples from Ballester-Lozano et al. (152).

The combination of conventional and different -omic approaches (functional genomics, proteomics, metabolomics, metagenomics) have been gaining acceptance as an option to assess the nutritionally and environmentally mediated effects on the growth performance, metabolic homeostasis, stress responsiveness and health condition of farmed fishes. However, at the present stage, a reliable diagnostic should combine measures of conventional biomarkers (e.g., data on blood biochemistry and histopathological scoring) with molecular signatures of the different components of the Gh/Igf system in addition to other related markers of growth and cell differentiation and proliferation, protein breakdown, protein folding and assembly, inflammatory/anti-inflammatory response, energy sensing, OXPHOS, mitochondrial respiration uncoupling, and lipolytic/lipogenic activity. In our hands, a key point for the simultaneous gene expression profiling of all the genes included in our growth PCR-array is the operation of the analytical platform by handling robots, resulting in a minimal variation between technical replicates. By means of multivariate analysis, this offers the possibility to identify at a high level of confidence the most responsive tissues and biomarkers in animals facing a given stressful rearing condition. This is exemplified herein by comparing the molecular signatures of liver and skeletal muscle of fishes with signs of nutrient deficiencies in n-3 LC-PUFA, phosphorus, or vitamins (Figures 11, 12). These three types of nutrient deficiencies were chosen because they are considered the most constraining factors of FM/FO replacement by alternative plant ingredients in marine farmed fishes. Using this approach, discriminant analysis (PLS-DA) is able to explain more than 93% of the variance (R) and to predict more than 72% of the total variance (Q). Thus, from this meta-analysis, it is conclusive that the liver tissue is especially responsive to deficiencies in essential fatty acids or phosphorus, whereas skeletal muscle is emerging as the main target tissue for the diagnosis of vitamin deficiencies. This is supported by variable importance (VIP) analysis, which highlights the different contribution of the 73–84 genes analyzed in our growth-arrays. This is just one example of what can be done with this type of approach, helping to establish the normal range of variance of highly informative biomarkers as a function of developmental stage and nutritional background. This procedure is based on the ARRAINA-derived biomarkers (229), and current research is taking advantage of this knowledge within the PerformFISH and GAIN H2020 EU Projects to validate, at the pilot scale and farm levels, new rearing systems and diet formulations for European aquaculture intensification. How all this is affected by nutrition and genome interactions is, however, a major challenge in efficiently managing aquaculture breeding programs and producing more robust farmed fishes, fed sustainable diets in a changing environment. In this regard, integrative studies on fish endocrinology can help to establish the best phenotype and the normal range of reference for different growth-promoting factors in animals with different nutritional and environmental backgrounds, allowing us to re-evaluate the nutritional status and nutrient requirements. How this can also include other criteria, such as functional microbiota, requires more research, but the endocrine system can help explain the now-emerging distal effects of intestinal microbiota on productive traits other than those more directly related to intestinal health. For instance, a growing body of evidence is pointing out that microbiota modulates host circulating Igf-i levels (230), a feature that seems to be conserved in fishes (231), and probably mediated by microbiota production of short-chain fatty acids. However, studies in fishes relating the composition of the core microbiota to a wide range of endogenous and exogenous factors are still in their infancy.

## Author contributions

JP-S: writing, reviewing, and conception; PS-M, FN-C, JM-S, EP, AB-N, and LB-P: writing; JC-G: writing and reviewing.

### Conflict of interest statement

The authors declare that the research was conducted in the absence of any commercial or financial relationships that could be construed as a potential conflict of interest.
