# Supplementary material for: Somatotropic Axis Regulation Unravels the Differential Effects of Nutritional and Environmental Factors in Growth Performance of Marine Farmed Fishes
Source: Front Endocrinol (Lausanne). 2018 Nov 27;9:687. doi: 10.3389/fendo.2018.00687 (PMC6277588; doi:10.3389/fendo.2018.00687)
Supplement: Supplementary file 1 [file Table_1.DOC]

**Supplemental Table S1.** Effect of nutrient deficiencies on the relative mRNA expression of growth-related genes in liver of gilthead sea bream fed to visual satiety from May to July (13 weeks). Data are the mean±SEM of 6 fishes. All data are referenced to the expression level of *igf-iir* of control fishes (CTRL diet) with an arbitrarily assigned value of 1. Different superscript letters in each row indicate significant differences among dietary treatments (P < 0.05; ANOVA followed by Student-Newman-Keuls test).

|  | CTRL | SAA | n-3  LC-PUFA | PL | Pi | Min | Vit | P-value^1^ |
| --- | --- | --- | --- | --- | --- | --- | --- | --- |
| *ghr-i* | 22.04±2.56^a^ | 18.45±2.46^a^ | 9.93±0.98^b^ | 22.98±3.1^a^ | 9.04±0.98^b^ | 19.44±2.67^a^ | 17.63±2.32^a^ | <0.001 |
| *ghr-ii* | 12.53±2.21 | 12.4±1.97 | 8.45±1.23 | 10.76±2.19 | 12.07±1.83 | 16.02±1.44 | 16.94±2.35 | 0.068 |
| *igf-i* | 78.57±5.77^a^ | 66.99±8.31^a^ | 30.75±2.29^b^ | 65.31±5.62^a^ | 26.21±4.65^b^ | 70.1±4.88^a^ | 60.97±7.82^a^ | <0.001 |
| *igf-ii* | 28.94±2.98^ab^ | 23.83±4.08^ab^ | 18.3±2.81^b^ | 23.43±5.32^ab^ | 7.56±1.89^c^ | 25.28±4.63^ab^ | 38.22±3.59^a^ | <0.001 |
| *igfb-1a* | 0.14±0.01 | 0.12±0.02 | 0.11±0.01 | 0.18±0.05 | 0.15±0.02 | 0.17±0.03 | 0.21±0.02 | 0.070 |
| *igfbp-2b* | 17.41±1.54^ab^ | 17.07±2.35^ab^ | 12.21±0.34^b^ | 16.05±0.9^ab^ | 16.46±1.44^ab^ | 19.4±1.86^a^ | 16.67±1.88^ab^ | 0.014 |
| *igfbp-4* | 7.56±0.69^a^ | 6.25±0.87^ab^ | 4.72±0.18^b^ | 6.85±0.49^ab^ | 2.48±0.27^c^ | 5.65±0.75^ab^ | 6.64±0.87^ab^ | <0.001 |
| *igfals* | 159.5±22.08^a^ | 141.3±23.15^ab^ | 99.55±3.27^b^ | 138.6±15.67^ab^ | 47.61±5.38^c^ | 176.35±17.58^a^ | 173.6±12.36^a^ | <0.001 |
| *insr* | 4.18±0.56 | 3.51±0.45 | 3.12±0.38 | 3.94±0.29 | 3.37±0.27 | 3.93±0.27 | 4.28±0.37 | 0.283 |
| *igf-ira* | 0.24±0.02 | 0.22±0.02 | 0.23±0.03 | 0.23±0.02 | 0.24±0.03 | 0.22±0.02 | 0.25±0.02 | 0.963 |
| *igf-iir* | 1.01±0.06 | 0.98±0.07 | 1.11±0.06 | 1.15±0.06 | 1.11±0.11 | 1.04±0.11 | 1.1±0.09 | 0.820 |
| *mef2a* | 2.88±0.31 | 2.83±0.2 | 2.78±0.27 | 3.27±0.27 | 3.3±0.25 | 3.07±0.33 | 3.05±0.28 | 0.750 |
| *mef2c* | 0.2±0.02 | 0.18±0.02 | 0.17±0.01 | 0.16±0.03 | 0.21±0.02 | 0.18±0.02 | 0.16±0.01 | 0.579 |
| *vim* | 0.37±0.05^ab^ | 0.41±0.05^ab^ | 0.23±0.03^a^ | 0.38±0.05^ab^ | 0.35±0.05^ab^ | 0.46±0.07^b^ | 0.40±0.04^ab^ | 0.156 |
| *pcna* | 5.49±0.53^ab^ | 4.89±0.76^ab^ | 3.03±0.47^a^ | 6.15±1.23^ab^ | 4.43±0.95^ab^ | 4.61±0.94^ab^ | 7.61±0.77^b^ | 0.020 |
| *met* | 4.7±0.38^ab^ | 3.89±0.4^a^ | 5.03±0.61^ab^ | 5.53±0.64^ab^ | 4.9±0.53^ab^ | 6.81±0.76^b^ | 6.95±0.59^b^ | 0.004 |
| *capn1* | 4.06±0.51^a^ | 4.06±0.29^a^ | 2.37±0.3^b^ | 4.13±0.66^a^ | 2.15±0.28^b^ | 2.99±0.24^ab^ | 3.29±0.37^ab^ | 0.002 |
| *capn2* | 1.04±0.07 | 1.04±0.11 | 1.22±0.17 | 1.22±0.14 | 1.59±0.27 | 0.99±0.14 | 1.17±0.22 | 0.275 |
| *capn3* | 0.08±0.01 | 0.14±0.02 | 0.12±0.02 | 0.12±0.01 | 0.08±0.01 | 0.1±0.02 | 0.11±0.01 | 0.063 |
| *cast* | 4.24±0.37 | 3.29±0.46 | 4.59±0.39 | 4.35±0.36 | 3.47±0.35 | 3.69±0.35 | 4.74±0.22 | 0.035 |
| *ctsb* | 23.47±1.08 | 23.51±5.76 | 26.7±2.78 | 26.35±2.91 | 27.41±4.44 | 26.18±4.03 | 26.35±3.64 | 0.988 |
| *ctsd* | 3.15±0.29^a^ | 1.86±0.28^ab^ | 1.41±0.17^b^ | 2.04±0.44^ab^ | 3.02±0.43^a^ | 1.81±0.33^ab^ | 1.80±0.29^ab^ | 0.006 |
| *ctsl* | 58.37±8.65^abc^ | 42.55±7.96^ab^ | 68.88±6.24^bc^ | 75.85±10.13^c^ | 30.42±3.73^a^ | 48.40±5.88^abc^ | 58.57±6.99^abc^ | 0.001 |
| *ctss* | 1.67±0.25 | 2.44±0.27 | 2.27±0.2 | 2.01±0.08 | 1.98±0.39 | 2.17±0.24 | 2.46±0.27 | 0.443 |
| *psmd4* | 1.81±0.12 | 1.92±0.29 | 1.46±0.04 | 2.25±0.41 | 1.94±0.23 | 1.61±0.17 | 1.84±0.11 | 0.323 |
| *psd12* | 2.5±0.18 | 2.44±0.39 | 2.44±0.16 | 4.03±0.83 | 3.09±0.34 | 2.51±0.35 | 2.84±0.12 | 0.068 |

**Supplemental Table S1 (continued).**

|  | CTRL | SAA | n-3  LC-PUFA | PL | Pi | Min | Vit | P-value^1^ |
| --- | --- | --- | --- | --- | --- | --- | --- | --- |
| *psma5* | 2.13±0.27 | 2.1±0.56 | 1.72±0.08 | 3.28±1.1 | 2.55±0.43 | 1.6±0.31 | 1.91±0.14 | 0.262 |
| *psmb1a* | 6.12±0.82 | 5.13±0.85 | 5.07±0.23 | 8.98±3.69 | 6.24±0.86 | 5.12±0.87 | 5.48±0.14 | 0.533 |
| *uchl3* | 1.64±0.09 | 1.29±0.26 | 1.55±0.15 | 1.83±0.44 | 2.45±0.65 | 1.36±0.2 | 1.43±0.16 | 0.302 |
| *ube2a* | 1.91±0.17 | 1.45±0.17 | 1.72±0.05 | 1.98±0.28 | 2.35±0.29 | 1.91±0.21 | 1.95±0.17 | 0.143 |
| *ube2d2* | 5.68±0.26 | 5.32±0.48 | 5.1±0.22 | 5.75±0.42 | 6.22±0.46 | 6.21±0.7 | 5.92±0.28 | 0.464 |
| *ube2l3* | 10.55±0.61 | 9.43±1.13 | 8.68±0.31 | 11.56±1.43 | 11.6±1.35 | 9.77±0.96 | 10.67±0.52 | 0.356 |
| *ube2n* | 3.12±0.4 | 2.66±0.39 | 2.87±0.18 | 2.92±0.65 | 3.24±0.45 | 2.69±0.41 | 3.03±0.24 | 0.941 |
| *cul2* | 0.94±0.06 | 0.92±0.11 | 0.9±0.05 | 1.07±0.15 | 1.16±0.23 | 0.93±0.11 | 0.9±0.04 | 0.715 |
| *cul3* | 0.96±0.08 | 0.82±0.1 | 0.8±0.06 | 0.99±0.1 | 0.98±0.09 | 0.83±0.1 | 0.85±0.06 | 0.501 |
| *cul5* | 0.41±0.03 | 0.45±0.06 | 0.41±0.04 | 0.5±0.07 | 0.44±0.04 | 0.44±0.05 | 0.48±0.02 | 0.812 |
| *mthsp10* | 6.9±0.61 | 7.29±0.97 | 5.42±0.26 | 5.43±0.75 | 6.99±0.92 | 7.32±1.03 | 5.22±0.77 | 0.245 |
| *mthsp30* | 0.06±0.01 | 0.06±0 | 0.07±0.01 | 0.06±0.01 | 0.06±0.01 | 0.06±0.02 | 0.06±0.01 | 0.994 |
| *mthsp60* | 2.3±0.26 | 2.49±0.35 | 1.86±0.11 | 2.04±0.25 | 2.57±0.22 | 3.08±0.4 | 2.56±0.45 | 0.212 |
| *mthsp70* | 3.13±0.34 | 3.27±0.5 | 2.71±0.32 | 3.31±0.47 | 5.37±1.19 | 4.09±0.37 | 3.86±0.56 | 0.127 |
| *hsp90β* | 192.44±14.25 | 164.35±13.5 | 181.49±7.07 | 201.97±24.52 | 218.17±34.41 | 199.52±14.75 | 169.95±11.31 | 0.528 |
| *grp-170* | 8.33±0.83^ab^ | 9.30±1.65^ab^ | 4.88±0.49^a^ | 10.86±2.78^ab^ | 11.45±1.26^ab^ | 12.1±1.81^b^ | 9.86±0.74^ab^ | 0.066 |
| *grp-94* | 25.95±2.53^ab^ | 20.56±4.77^a^ | 19.68±1.4^a^ | 30.07±5.8^ab^ | 18.12±1.74^a^ | 38.34±5.82^b^ | 27.09±4.11^ab^ | 0.013 |
| *der-1* | 9.67±0.87^ab^ | 8.14±1.20^ab^ | 5.84±0.17^a^ | 10.83±2.08^b^ | 7.47±0.61^ab^ | 10.38±1.42^ab^ | 10.29±0.63^ab^ | 0.025 |
| *il-1β* | 0.01±0.01 | 0.03±0.01 | 0.02±0.01 | 0.05±0.04 | 0.01±0.01 | 0.01±0.01 | 0.01±0.01 | 0.627 |
| *il-1r1* | 3.32±0.62^ab^ | 2.6±0.24^ab^ | 2.65±0.18^ab^ | 4.58±0.60^b^ | 1.75±0.18^a^ | 3.44±0.52^ab^ | 4.75±1.00^b^ | 0.005 |
| *il-1r2* | 0.01±0 | 0.03±0.02 | 0.05±0.02 | 0.04±0.03 | 0.01±0 | 0.02±0 | 0.02±0.01 | 0.146 |
| *il-6* | 0.01±0 | 0.01±0 | 0.01±0 | 0.01±0 | 0.01±0 | 0.01±0 | 0.01±0 | 0.615 |
| *il-6ra* | 9.39±0.93 | 7.06±0.45 | 10.8±0.79 | 11.51±2.12 | 8.03±0.74 | 11.71±2.04 | 10.56±0.73 | 0.087 |
| *il-6rb* | 8.25±0.67^ab^ | 6.86±0.74^abc^ | 5.41±0.37^c^ | 8.88±1.01^a^ | 5.81±0.42^bc^ | 8.21±0.80^ab^ | 9.41±0.66^a^ | 0.001 |
| *il-8* | 0.01±0 | 0.03±0.01 | 0.03±0 | 0.03±0.02 | 0.02±0.01 | 0.01±0 | 0.02±0.01 | 0.692 |
| *il-8ra* | 0.11±0.02 | 0.21±0.07 | 0.11±0.04 | 0.17±0.04 | 0.08±0.02 | 0.19±0.06 | 0.17±0.02 | 0.208 |
| *il-10* | 0.04±0.01 | 0.05±0.01 | 0.03±0.01 | 0.03±0.01 | 0.03±0.01 | 0.04±0.01 | 0.03±0 | 0.505 |
| *il-10ra* | 0.1±0.01 | 0.13±0.02 | 0.14±0.02 | 0.09±0.01 | 0.11±0.01 | 0.1±0.02 | 0.11±0.01 | 0.331 |
| *il-10rb* | 4.96±0.93 | 4.45±0.64 | 3.95±0.23 | 4.82±0.37 | 4.2±0.44 | 4.97±0.57 | 5.02±0.45 | 0.720 |

**Supplemental Table S1 (continued).**

|  | CTRL | SAA | n-3  LC-PUFA | PL | Pi | Min | Vit | P-value^1^ |
| --- | --- | --- | --- | --- | --- | --- | --- | --- |
| *tnf-α* | 0.14±0.02 | 0.12±0.02 | 0.13±0.02 | 0.17±0.01 | 0.11±0.01 | 0.14±0.01 | 0.13±0.01 | 0.325 |
| *tradd* | 0.96±0.06 | 0.83±0.11 | 0.92±0.04 | 0.91±0.09 | 0.87±0.05 | 0.93±0.13 | 1.02±0.04 | 0.714 |
| *sirt1* | 0.58±0.04 | 0.64±0.11 | 0.56±0.02 | 0.65±0.05 | 0.63±0.04 | 0.54±0.06 | 0.58±0.03 | 0.715 |
| *sirt2* | 1.83±0.08 | 1.56±0.20 | 1.54±0.07 | 1.94±0.15 | 1.74±0.15 | 1.73±0.21 | 1.71±0.09 | 0.528 |
| *sirt3* | 0.22±0.02 | 0.28±0.03 | 0.2±0.02 | 0.3±0.04 | 0.2±0.02 | 0.26±0.04 | 0.3±0.03 | 0.060 |
| *sirt4* | 0.10±0.01^a^ | 0.10±0.01^a^ | 0.11±0.01^ab^ | 0.12±0.02^ab^ | 0.15±0.01^b^ | 0.12±0.01^ab^ | 0.10±0.01^a^ | 0.017 |
| *sirt5* | 1.7±0.08 | 1.82±0.16 | 1.59±0.15 | 1.92±0.15 | 1.81±0.15 | 1.48±0.14 | 1.96±0.17 | 0.266 |
| *pgc1α* | 0.45±0.14 | 0.58±0.08 | 0.49±0.1 | 0.65±0.08 | 0.88±0.15 | 1.03±0.1 | 0.78±0.19 | 0.042 |
| *cpt1a* | 2.41±0.14^ab^ | 2.41±0.27^ab^ | 5.29±0.69^c^ | 3.16±0.53^ab^ | 1.73±0.20^a^ | 3.33±0.38^ab^ | 3.50±0.39^b^ | <0.001 |
| *cs* | 5.58±0.26 | 5.85±0.94 | 6.81±0.72 | 5.51±0.44 | 7.49±0.86 | 6.17±0.47 | 5.54±0.45 | 0.221 |
| *nd2* | 180.27±12.73 | 131.02±17.47 | 148.43±11.31 | 187.7±32.25 | 264.63±48.47 | 191.83±31.07 | 162.34±8.53 | 0.050 |
| *ndufaf2* | 1.6±0.12 | 1.46±0.14 | 1.37±0.1 | 1.84±0.17 | 1.78±0.15 | 1.61±0.14 | 1.76±0.1 | 0.182 |
| *coxi* | 463.51±43.36 | 305.52±44.29 | 405.67±38.81 | 366.53±41.55 | 521.73±86.17 | 359.45±45.96 | 509.48±50.46 | 0.096 |
| *sco1* | 0.36±0.01^a^ | 0.43±0.06^a^ | 0.40±0.03^a^ | 0.47±0.09^a^ | 0.65±0.06^b^ | 0.40±0.05^a^ | 0.42±0.04^a^ | 0.009 |
| *ucp1* | 81.02±3.9^ab^ | 61.22±10.60^a^ | 90.54±10.03^ab^ | 77.84±9.07^ab^ | 107.3±12.64^b^ | 67.12±6.27^a^ | 57.62±7.99^a^ | 0.005 |
| *lxrα* | 5.48±0.57 | 3.73±0.74 | 5.58±0.24 | 4.6±0.39 | 5.24±0.6 | 4.56±0.63 | 4.79±0.69 | 0.358 |
| *pparα* | 18.17±1.74 | 15.83±2.48 | 14.57±0.62 | 19.4±2.55 | 22.67±3.55 | 19.6±1.74 | 17.42±1.39 | 0.225 |
| *pparγ* | 3.66±0.59 | 4.11±1.00 | 2.93±0.17 | 3.5±0.13 | 3.8±0.32 | 3.82±0.43 | 3.7±0.44 | 0.805 |

^1^Result values from one-way analysis of variance
