# Supplementary material for: Somatotropic Axis Regulation Unravels the Differential Effects of Nutritional and Environmental Factors in Growth Performance of Marine Farmed Fishes
Source: Front Endocrinol (Lausanne). 2018 Nov 27;9:687. doi: 10.3389/fendo.2018.00687 (PMC6277588; doi:10.3389/fendo.2018.00687)
Supplement: Supplementary file 2 [file Table_2.DOC]

**Supplemental Table S2.** Effect of nutrient deficiencies on the relative mRNA expression of growth-related genes in skeletal muscle of gilthead sea bream fed to visual satiety from May to July (13 weeks). Data are the mean±SEM of 6 fishes. All data are referenced to the expression level of *igf-iir* of control fishes (CTRL diet) with an arbitrarily assigned value of 1. Different superscript letters in each row indicate significant differences among dietary treatments (P < 0.05; ANOVA followed by Student-Newman-Keuls test).

|  | CTRL | SAA | n-3  LC-PUFA | PL | Pi | Min | Vit | P-value^1^ |
| --- | --- | --- | --- | --- | --- | --- | --- | --- |
| *ghr-i* | 6.96±0.72^ab^ | 6.88±0.87^ab^ | 5.60±0.31^a^ | 10.02±1.31^b^ | 8.20±1.11^ab^ | 8.13±1.03^ab^ | 7.17±0.94^ab^ | 0.050 |
| *ghr-ii* | 2.00±0.24^a^ | 3.47±0.64^ab^ | 6.13±0.63^ab^ | 3.25±0.84^ab^ | 8.05±1.61^b^ | 4.04±0.92^ab^ | 6.61±2.29^ab^ | 0.006 |
| *igf-i* | 0.89±0.06 | 0.79±0.06 | 0.70±0.05 | 0.90±0.12 | 0.68±0.06 | 0.92±0.13 | 0.63±0.07 | 0.127 |
| *igf-ii* | 3.92±0.48^a^ | 4.12±0.31^a^ | 3.65±0.19^a^ | 3.65±0.27^a^ | 3.85±0.33^a^ | 3.77±0.15^a^ | 5.14±0.40^b^ | 0.038 |
| *igfbp-1a* | 0.58±0.12 | 0.34±0.07 | 0.35±0.02 | 0.47±0.11 | 0.39±0.04 | 0.31±0.04 | 0.49±0.04 | 0.139 |
| *igfbp-3* | 8.01±0.84^a^ | 8.53±0.69^a^ | 7.08±0.98^a^ | 8.47±1.61^a^ | 15.80±1.62^b^ | 10.31±1.42^a^ | 8.15±1.24^a^ | <0.001 |
| *igfbp-4* | 1.11±0.12 | 0.82±0.10 | 0.72±0.09 | 0.88±0.22 | 0.67±0.09 | 0.78±0.09 | 0.78±0.12 | 0.262 |
| *igfbp-5b* | 9.83±0.94 | 8.50±0.76 | 7.50±0.87 | 9.76±1.38 | 8.50±1.09 | 8.19±0.52 | 7.57±0.52 | 0.424 |
| *igfbp-6b* | 0.91±0.10 | 0.80±0.04 | 0.58±0.13 | 0.75±0.13 | 0.76±0.08 | 0.82±0.08 | 1.04±0.08 | 0.094 |
| *insr* | 1.94±0.16 | 1.96±0.17 | 2.37±0.19 | 2.13±0.23 | 2.65±0.25 | 2.19±0.17 | 1.92±0.2 | 0.140 |
| *igf-ira* | 1.38±0.10 | 1.30±0.15 | 1.41±0.09 | 1.66±0.09 | 1.21±0.12 | 1.41±0.11 | 1.52±0.18 | 0.270 |
| *igf-iir* | 1.02±0.07^ac^ | 0.88±0.07^abc^ | 0.79±0.05^ab^ | 1.06±0.09^ac^ | 0.69±0.07^b^ | 0.88±0.07^abc^ | 1.12±0.07^c^ | 0.001 |
| *myod1* | 18.04±2.06 | 17.88±1.54 | 18.64±1.4 | 23.27±1.14 | 23.81±2.52 | 20.02±1.60 | 23.62±2.66 | 0.087 |
| *myod2* | 9.47±0.84^ab^ | 11.4±0.88^b^ | 10.92±1.38^b^ | 10.47±1.03^ab^ | 12.92±1.15^b^ | 10.28±0.86^ab^ | 6.81±0.37^a^ | 0.009 |
| *myf5* | 1.68±0.08 | 1.44±0.13 | 1.39±0.10 | 1.22±0.07 | 1.61±0.10 | 1.50±0.12 | 1.52±0.11 | 0.081 |
| *myf6* | 1.37±0.22 | 1.22±0.15 | 1.24±0.11 | 1.38±0.11 | 1.34±0.12 | 1.12±0.07 | 1.08±0.11 | 0.541 |
| *mstn* | 6.43±1.23^a^ | 8.01±1.93^a^ | 7.02±1.04^a^ | 5.61±0.84^a^ | 20.60±2.05^b^ | 8.25±1.22^a^ | 12.02±2.02^a^ | <0.001 |
| *mef2a* | 35.83±3.83^a^ | 32.75±4.17^a^ | 51.95±4.13^b^ | 40.00±4.30^a^ | 36.65±3.31^a^ | 30.92±3.65^a^ | 32.55±3.98^a^ | 0.011 |
| *mef2c* | 12.28±1.20 | 10.68±1.02 | 14.32±0.93 | 12.50±0.55 | 12.48±0.65 | 11.15±0.43 | 12.49±0.77 | 0.097 |
| *fst* | 1.67±0.22^a^ | 1.22±0.14^ab^ | 0.92±0.13^b^ | 0.97±0.10^b^ | 1.47±0.14^ab^ | 1.44±0.17^ab^ | 1.62±0.08^a^ | 0.004 |
| *cav3* | 81.83±7.70 | 77.51±9.76 | 82.54±5.74 | 83.68±6.88 | 92.45±7.20 | 81.44±3.15 | 85.56±9.59 | 0.886 |
| *des* | 259.8±33.90 | 238.0±33.18 | 232.7±21.44 | 255.6±21.21 | 191.59±20.8 | 226.01±18.77 | 270.25±40.82 | 0.545 |
| *vim* | 1.63±0.20 | 1.68±0.25 | 1.18±0.09 | 1.48±0.13 | 1.49±0.05 | 1.55±0.14 | 1.35±0.2 | 0.350 |
| *cdh15* | 2.31±0.22 | 2.13±0.21 | 2.16±0.25 | 1.86±0.21 | 2.16±0.33 | 2.13±0.18 | 1.97±0.12 | 0.860 |
| *pcna* | 3.06±0.32 | 3.59±0.37 | 2.89±0.33 | 3.18±0.61 | 3.20±0.37 | 3.42±0.29 | 3.21±0.16 | 0.875 |
| *pax7* | 0.23±0.02 | 0.17±0.01 | 0.18±0.03 | 0.19±0.02 | 0.21±0.03 | 0.18±0.02 | 0.16±0.02 | 0.257 |
| *met* | 0.47±0.03 | 0.37±0.03 | 0.36±0.05 | 0.38±0.04 | 0.45±0.03 | 0.39±0.01 | 0.45±0.03 | 0.127 |

**Supplemental Table S2 (continued).**

|  | CTRL | SAA | n-3  LC-PUFA | PL | Pi | Min | Vit | P-value^1^ |
| --- | --- | --- | --- | --- | --- | --- | --- | --- |
| *capn1* | 4.80±0.33 | 4.70±0.49 | 4.18±0.43 | 4.75±0.34 | 4.35±0.38 | 4.58±0.37 | 5.21±0.38 | 0.641 |
| *capn2* | 6.95±0.59 | 7.08±0.83 | 4.83±0.40 | 6.43±0.70 | 5.96±0.59 | 7.13±0.70 | 6.94±0.45 | 0.140 |
| *capn3* | 13.63±1.93^ab^ | 10.18±1.29^a^ | 12.18±1.09^ab^ | 15.74±2.37^b^ | 10.77±1.44^ab^ | 9.00±1.05^ab^ | 15.92±1.26^b^ | 0.017 |
| *cast* | 14.55±1.06^a^ | 12.79±1.06^a^ | 17.65±1.32^ab^ | 14.23±1.42^a^ | 19.95±1.15^b^ | 15.15±1.08^a^ | 17.05±1.22^ab^ | 0.003 |
| *ctsb* | 7.22±0.26^a^ | 9.05±2.45^a^ | 6.89±0.68^a^ | 6.67±0.40^a^ | 6.32±0.38^a^ | 9.97±2.31^a^ | 21.2±2.72^b^ | <0.001 |
| *ctsd* | 1.01±0.08^a^ | 0.89±0.07^a^ | 0.92±0.05^a^ | 0.96±0.07^a^ | 1.16±0.08^a^ | 0.90±0.07^a^ | 1.60±0.13^b^ | <0.001 |
| *ctsl* | 15.26±2.15 | 12.15±1.26 | 11.66±0.80 | 13.46±1.09 | 15.10±1.22 | 13.77±0.77 | 15.01±1.01 | 0.281 |
| *ctss* | 1.94±0.31^a^ | 1.97±0.47^a^ | 1.61±0.16^a^ | 1.84±0.33^a^ | 1.63±0.10^a^ | 2.42±0.53^a^ | 7.85±1.66^b^ | <0.001 |
| *psmd4* | 2.61±0.14 | 2.45±0.22 | 2.48±0.21 | 2.95±0.14 | 2.39±0.20 | 2.23±0.15 | 2.83±0.09 | 0.066 |
| *psd12* | 7.85±0.55 | 7.21±1.33 | 6.51±0.64 | 7.46±0.82 | 7.26±0.66 | 5.74±0.51 | 8.11±0.27 | 0.285 |
| *psma5* | 4.13±0.26 | 4.05±0.50 | 4.11±0.51 | 4.72±0.60 | 4.20±0.34 | 3.40±0.32 | 4.87±0.39 | 0.284 |
| *psmb1a* | 8.91±0.69 | 6.72±0.49 | 7.37±0.49 | 8.61±1.11 | 8.27±0.70 | 7.01±0.68 | 8.3±0.46 | 0.218 |
| *uchl3* | 4.92±0.60^ab^ | 3.69±0.15^a^ | 4.54±0.50^ab^ | 4.71±0.43^ab^ | 5.12±0.37^ab^ | 3.69±0.32^a^ | 5.77±0.34^b^ | 0.021 |
| *ube2a* | 4.91±0.49 | 4.05±0.66 | 4.20±0.33 | 5.06±0.34 | 5.00±0.59 | 4.15±0.37 | 4.73±0.34 | 0.499 |
| *ube2d2* | 2.70±0.21 | 2.57±0.25 | 2.38±0.20 | 2.53±0.24 | 2.35±0.15 | 2.59±0.13 | 2.77±0.16 | 0.691 |
| *ube2l3* | 23.33±1.86 | 19.60±1.27 | 22.26±2.36 | 24.22±1.75 | 22.18±1.67 | 20.56±1.49 | 21.06±1.13 | 0.552 |
| *ube2n* | 17.67±1.52 | 16.36±1.54 | 17.95±2.66 | 19.03±1.82 | 17.37±1.13 | 15.29±1.25 | 16.13±0.84 | 0.751 |
| *cul2* | 2.27±0.16 | 2.07±0.27 | 2.10±0.16 | 2.52±0.14 | 2.17±0.18 | 1.88±0.10 | 2.13±0.19 | 0.286 |
| *cul3* | 3.08±0.29 | 2.94±0.32 | 2.85±0.18 | 3.46±0.35 | 3.45±0.29 | 2.69±0.08 | 2.78±0.14 | 0.192 |
| *cul5* | 0.71±0.04 | 0.79±0.13 | 0.75±0.06 | 0.87±0.09 | 0.86±0.07 | 0.64±0.03 | 0.76±0.05 | 0.364 |
| *mthsp10* | 8.34±1.57 | 5.73±0.94 | 7.36±0.83 | 9.27±1.93 | 5.85±0.79 | 6.07±0.45 | 8.69±0.98 | 0.203 |
| *mthsp60* | 2.97±0.47 | 2.22±0.38 | 2.72±0.34 | 3.22±0.89 | 2.51±0.31 | 2.06±0.17 | 3.11±0.21 | 0.429 |
| *mthsp70* | 6.36±0.80 | 4.69±0.39 | 5.60±0.41 | 6.31±0.95 | 6.70±0.65 | 4.92±0.36 | 6.91±0.55 | 0.097 |
| *hsp90α* | 117.6±26.02 | 81.00±10.70 | 124.1±9.90 | 101.8±14.71 | 101.8±8.85 | 84.9±6.91 | 132.21±12.84 | 0.154 |
| *hsp90β* | 44.20±1.21 | 40.54±3.22 | 41.87±3.56 | 45.43±6.23 | 43.11±2.58 | 46.2±3.49 | 56.66±4 | 0.088 |
| *grp-170* | 1.89±0.12^ab^ | 1.93±0.25^ab^ | 1.71±0.14^a^ | 1.92±0.22^ab^ | 1.92±0.18^ab^ | 1.81±0.15^ab^ | 2.53±0.15^b^ | 0.050 |
| *grp-94* | 4.91±0.86 | 4.62±0.45 | 5.25±0.87 | 4.32±0.39 | 4.59±0.43 | 5.01±0.47 | 6.01±1.06 | 0.741 |
| *der-1* | 9.71±0.64 | 9.49±0.89 | 9.43±0.87 | 10.58±0.84 | 10.33±0.90 | 8.96±0.47 | 11.81±0.48 | 0.168 |
| *il-1β* | 0.10±0.02^ab^ | 0.04±0.01^a^ | 0.06±0.01^ab^ | 0.17±0.07^b^ | 0.10±0.01^ab^ | 0.06±0.01^ab^ | 0.11±0.01^ab^ | 0.049 |

**Supplemental Table S2 (continued).**

|  | CTRL | SAA | n-3  LC-PUFA | PL | Pi | Min | Vit | P-value^1^ |
| --- | --- | --- | --- | --- | --- | --- | --- | --- |
| *il-1r1* | 0.75±0.06^a^ | 0.68±0.05^a^ | 0.63±0.04^a^ | 0.73±0.05^a^ | 0.68±0.06^a^ | 0.76±0.06^a^ | 1.29±0.13^b^ | <0.001 |
| *il-1r2* | 0.02±0.01^a^ | 0.01±0.01^a^ | 0.01±0.01^a^ | 0.01±0.01^a^ | 0.01±0.01^a^ | 0.02±0.01^a^ | 0.09±0.03^b^ | <0.001 |
| *il-6* | 0.03±0.01 | 0.03±0.01 | 0.03±0.01 | 0.02±0.01 | 0.03±0.01 | 0.02±0.01 | 0.05±0.02 | 0.287 |
| *il-6ra* | 0.54±0.08 | 0.37±0.03 | 0.37±0.02 | 0.52±0.05 | 0.43±0.03 | 0.40±0.03 | 0.58±0.09 | 0.028 |
| *il-6rb* | 3.03±0.22^a^ | 3.17±0.35^a^ | 3.23±0.19^a^ | 3.91±0.35^ab^ | 4.03±0.46^ab^ | 3.37±0.26^a^ | 4.77±0.22^b^ | 0.002 |
| *il-8* | 0.06±0.02 | 0.03±0.01 | 0.03±0.01 | 0.12±0.09 | 0.03±0.01 | 0.06±0.01 | 0.07±0.01 | 0.492 |
| *il-10* | 0.05±0.01^a^ | 0.06±0.01^a^ | 0.05±0.01^a^ | 0.05±0.01^a^ | 0.05±0.01^a^ | 0.07±0.01^a^ | 0.15±0.02^b^ | <0.001 |
| *il-10ra* | 0.06±0.01^a^ | 0.05±0.01^a^ | 0.04±0.01^a^ | 0.06±0.01^a^ | 0.04±0.01^a^ | 0.06±0.03^a^ | 0.19±0.05^b^ | <0.001 |
| *il-10rb* | 0.81±0.08^a^ | 0.83±0.06^a^ | 0.86±0.06^a^ | 0.95±0.08^a^ | 0.97±0.08^a^ | 0.97±0.05^a^ | 1.42±0.10^b^ | <0.001 |
| *tnf-α* | 0.14±0.02 | 0.13±0.01 | 0.11±0.01 | 0.13±0.02 | 0.13±0.02 | 0.13±0.02 | 0.11±0.01 | 0.892 |
| *tradd* | 0.43±0.06 | 0.36±0.05 | 0.42±0.05 | 0.32±0.03 | 0.46±0.04 | 0.32±0.05 | 0.5±0.03 | 0.064 |
| *sirt1* | 0.75±0.06 | 0.74±0.06 | 0.73±0.05 | 0.77±0.06 | 0.83±0.07 | 0.79±0.06 | 0.84±0.06 | 0.807 |
| *sirt2* | 1.39±0.13 | 1.42±0.13 | 1.24±0.09 | 1.55±0.08 | 1.56±0.16 | 1.48±0.07 | 1.46±0.09 | 0.474 |
| *sirt3* | 0.18±0.02^a^ | 0.22±0.03^ab^ | 0.19±0.02^a^ | 0.21±0.03^ab^ | 0.17±0.02^a^ | 0.22±0.02^ab^ | 0.28±0.03^b^ | 0.041 |
| *sirt4* | 0.14±0.02 | 0.13±0.03 | 0.12±0.01 | 0.14±0.02 | 0.18±0.03 | 0.13±0.02 | 0.16±0.02 | 0.393 |
| *sirt5* | 1.99±0.23 | 2.19±0.2 | 2.22±0.22 | 2.91±0.24 | 2.27±0.25 | 2.01±0.17 | 2.26±0.26 | 0.127 |
| *pgc1α* | 0.42±0.10 | 0.45±0.16 | 0.54±0.12 | 0.79±0.26 | 0.33±0.12 | 0.32±0.03 | 0.30±0.08 | 0.176 |
| *cpt1a* | 7.98±1.88 | 6.34±1.03 | 7.36±0.25 | 9.27±1.19 | 6.72±0.85 | 7.04±0.62 | 6.65±0.88 | 0.574 |
| *cs* | 48.1±10.94 | 40.18±5.59 | 47.53±2.93 | 50.32±3.31 | 45.57±3.06 | 40.22±2.68 | 43.96±3.61 | 0.812 |
| *nd2* | 403.5±76.35 | 301.13±31.81 | 363.02±70.5 | 437.27±42.21 | 285.8±16.09 | 314.7±52.11 | 414.0±71.68 | 0.400 |
| *ndufaf2* | 2.29±0.29 | 2.27±0.32 | 2.05±0.14 | 2.52±0.18 | 2.34±0.13 | 2.14±0.11 | 2.38±0.23 | 0.768 |
| *coxi* | 824.0±112.6 | 642.83±95.42 | 704.02±73.29 | 776.15±50.83 | 862.9±45.64 | 553.3±37.06 | 744.6±61.82 | 0.073 |
| *sco1* | 0.36±0.07^a^ | 0.38±0.07^ab^ | 0.38±0.04^ab^ | 0.40±0.04^ab^ | 0.40±0.04^ab^ | 0.30±0.03^a^ | 0.60±0.08^b^ | 0.031 |
| *ucp2* | 0.85±0.14^ab^ | 0.60±0.12^a^ | 0.35±0.04^a^ | 0.48±0.06^a^ | 0.72±0.07^ab^ | 0.75±0.10^ab^ | 1.24±0.33^b^ | 0.006 |
| *ucp3* | 21.11±3.10 | 14.78±2.02 | 14.62±2.43 | 23.65±4.84 | 20.87±1.48 | 21.99±2.63 | 24.95±4.00 | 0.165 |
| *lxrα* | 1.34±0.12^a^ | 0.83±0.06^b^ | 0.95±0.08^bc^ | 0.90±0.06^bc^ | 1.21±0.05^ac^ | 0.99±0.06^bc^ | 1.29±0.07^a^ | <0.001 |
| *pparα* | 2.71±0.29 | 2.35±0.16 | 2.27±0.29 | 2.43±0.32 | 3.24±0.37 | 2.73±0.10 | 2.75±0.26 | 0.214 |
| *pparγ* | 1.71±0.19 | 1.35±0.29 | 0.97±0.08 | 1.52±0.40 | 1.34±0.12 | 1.60±0.21 | 1.45±0.20 | 0.319 |

^1^Result values from one-way analysis of variance
