# Supplementary material for: Somatotropic Axis Regulation Unravels the Differential Effects of Nutritional and Environmental Factors in Growth Performance of Marine Farmed Fishes
Source: Front Endocrinol (Lausanne). 2018 Nov 27;9:687. doi: 10.3389/fendo.2018.00687 (PMC6277588; doi:10.3389/fendo.2018.00687)
Supplement: Supplementary file 3 [file Table_3.DOCX]

**Supplementary Table S3.** Relative gene expression of growth-related genes in the liver of gilthead sea bream sampled in August. Data are the mean±SEM of 6-7 fishes. All data are referenced to the expression level of *igf-iir* of control fishes (D1 diet) with an arbitrarily assigned value of 1. Different superscript letters in each row indicate significant differences among dietary treatments (P < 0.05; ANOVA followed by Student-Newman-Keuls test).

|  | D1 | D2 | D3 | D4 | P-value^1^ |
| --- | --- | --- | --- | --- | --- |
| *ghr-i* | 19.41±0.99 | 18.53±2.07 | 19.63±2.12 | 20.12±3.42 | 0.898 |
| *ghr-ii* | 13.88±2.54 | 13.06±2.10 | 11.29±0.97 | 14.85±1.92 | 0.601 |
| *igf-i* | 68.1±8.65^a^ | 56.71±8.96^ab^ | 37.6±4.30^b^ | 47.21±4.07^ab^ | 0.018 |
| *igf-ii* | 28.09±5.30 | 30.36±2.76 | 20.83±4.96 | 23.21±2.85 | 0.335 |
| *igfbp-1a* | 0.11±0.03 | 0.11±0.01 | 0.14±0.01 | 0.10±0.01 | 0.212 |
| *igfbp-2b* | 17.13±2.19 | 14.59±1.41 | 13.55±1.18 | 13.96±1.49 | 0.452 |
| *igfbp-4* | 6.19±1.06 | 4.18±0.58 | 4.60±0.28 | 4.45±0.63 | 0.204 |
| *igfbp-5b* | 1.22±0.19 | 1.36±0.30 | 1.20±0.10 | 1.07±0.12 | 0.785 |
| *igfals* | 176.7±42.1 | 129.1±10.2 | 101.8±12.1 | 126.7±14.3 | 0.214 |
| *insr* | 3.05±0.11 | 2.25±0.26 | 2.68±0.25 | 2.56±0.14 | 0.101 |
| *igf-ira* | 0.25±0.02 | 0.19±0.01 | 0.24±0.01 | 0.21±0.01 | 0.057 |
| *igf-iir* | 1.04±0.06 | 0.90±0.06 | 0.95±0.05 | 0.96±0.07 | 0.469 |
| *mef2a* | 2.94±0.26 | 2.32±0.12 | 2.50±0.25 | 2.23±0.15 | 0.083 |
| *mef2c* | 0.28±0.02 | 0.25±0.02 | 0.35±0.05 | 0.25±0.02 | 0.136 |
| *pcna* | 8.73±1.13^a^ | 6.17±1.25^ab^ | 6.58±0.83^ab^ | 4.04±0.53^b^ | 0.017 |
| *met* | 4.11±0.59 | 3.81±0.21 | 3.82±0.59 | 3.47±0.31 | 0.785 |
| *capn1* | 2.94±0.26 | 2.27±0.25 | 3.16±0.33 | 2.24±0.23 | 0.051 |
| *capn2* | 1.24±0.17 | 1.04±0.04 | 1.37±0.10 | 1.18±0.08 | 0.212 |
| *capn3* | 0.09±0.02 | 0.08±0.01 | 0.13±0.03 | 0.08±0.01 | 0.292 |
| *cast* | 4.19±0.53 | 3.93±0.41 | 5.01±0.35 | 4.25±0.44 | 0.374 |
| *ctsb* | 30.61±2.51 | 33.9±3.15 | 37.67±3.65 | 33.74±4.19 | 0.593 |
| *ctsd* | 4.67±1.50 | 4.14±1.02 | 2.69±0.54 | 3.16±0.23 | 0.461 |
| *ctsl* | 86.43±10.19 | 77.26±10.64 | 77.91±9.76 | 74.30±12.19 | 0.875 |
| *ctss* | 2.12±0.12 | 2.38±0.35 | 2.65±0.32 | 2.44±0.28 | 0.635 |
| *psmd4* | 2.53±0.26 | 2.06±0.22 | 2.64±0.12 | 2.03±0.24 | 0.142 |
| *psd12* | 3.59±0.30 | 3.70±0.34 | 4.20±0.33 | 3.10±0.39 | 0.184 |
| *psma5* | 3.31±0.34 | 2.47±0.50 | 3.91±0.37 | 2.68±0.60 | 0.174 |
| *psmb1a* | 7.31±0.48 | 6.55±0.52 | 8.78±0.34 | 6.39±1.06 | 0.059 |
| *uchl3* | 2.07±0.14 | 1.50±0.17 | 2.29±0.19 | 1.82±0.30 | 0.107 |
| *ube2a* | 2.15±0.26 | 1.73±0.11 | 1.98±0.13 | 2.10±0.18 | 0.407 |
| *ube2d2* | 6.00±0.37 | 4.92±0.32 | 4.76±0.22 | 5.04±0.48 | 0.118 |
| *ube2l3* | 12.31±0.86 | 10.04±0.63 | 12.95±0.64 | 10.28±1.07 | 0.059 |
| *ube2n* | 3.83±0.15 | 3.23±0.26 | 3.86±0.07 | 3.56±0.42 | 0.373 |
| *cul2* | 1.06±0.09 | 0.91±0.06 | 1.06±0.06 | 0.94±0.07 | 0.331 |
| *cul3* | 0.99±0.08 | 0.86±0.07 | 0.98±0.06 | 0.81±0.05 | 0.140 |
| *cul5* | 0.43±0.03 | 0.42±0.04 | 0.44±0.03 | 0.38±0.03 | 0.564 |
| *mthsp10* | 7.30±0.61^a^ | 4.96±0.54^b^ | 6.65±0.54^ab^ | 5.17±0.53^b^ | 0.019 |
| *hsp30* | 0.04±0.01 | 0.05±0.01 | 0.07±0.02 | 0.05±0.01 | 0.417 |
| *mthsp60* | 2.39±0.31^a^ | 1.64±0.17^b^ | 1.83±0.08^b^ | 1.46±0.11^b^ | 0.011 |
| *mthsp70* | 4.91±0.44^a^ | 3.17±0.24^b^ | 2.90±0.22^b^ | 2.84±0.22^b^ | <0.001 |

**Supplementary Table S3. (continued)**

|  | D1 | D2 | D3 | D4 | P-value^1^ |
| --- | --- | --- | --- | --- | --- |
| *hsp90β* | 185.3±3.99^a^ | 134.4±7.32^b^ | 149.7±7.70^b^ | 133.67±4.38^b^ | <0.001 |
| *grp-170* | 6.10±0.62 | 5.80±0.76 | 6.36±0.42 | 4.85±0.44 | 0.268 |
| *grp-94* | 15.10±2.50 | 13.20±1.81 | 11.78±1.51 | 9.33±0.72 | 0.118 |
| *der-1* | 11.09±0.70 | 10.09±0.91 | 10.38±0.62 | 8.72±0.79 | 0.185 |
| *il-1β* | 0.01±0.01 | 0.01±0.01 | 0.03±0.01 | 0.01±0.01 | 0.325 |
| *il-1r1* | 2.36±0.26 | 2.46±0.28 | 3.50±0.80 | 3.63±0.94 | 0.206 |
| *il-1r2* | 0.01±0.01 | 0.01±0.01 | 0.04±0.02 | 0.01±0.01 | 0.053 |
| *il-6ra* | 8.42±1.03 | 6.65±0.51 | 8.48±1.44 | 8.50±0.80 | 0.495 |
| *il-6rb* | 7.40±0.42^a^ | 6.59±0.55^ab^ | 6.38±0.52^ab^ | 5.34±0.21^b^ | 0.020 |
| *il-8* | 0.03±0.01 | 0.03±0.01 | 0.04±0.01 | 0.03±0.01 | 0.463 |
| *il-8ra* | 0.15±0.03 | 0.14±0.04 | 0.22±0.07 | 0.15±0.04 | 0.576 |
| *il-10* | 0.05±0.01 | 0.04±0.01 | 0.04±0.01 | 0.03±0.01 | 0.613 |
| *il-10ra* | 0.20±0.05 | 0.16±0.02 | 0.19±0.05 | 0.14±0.01 | 0.518 |
| *il-10rb* | 5.83±0.42^a^ | 4.95±0.57^ab^ | 3.93±0.27^b^ | 4.52±0.54^ab^ | 0.048 |
| *tnf-α* | 0.13±0.01 | 0.11±0.01 | 0.11±0.01 | 0.11±0.01 | 0.555 |
| *tradd* | 1.10±0.05^a^ | 0.94±0.06^a^ | 1.41±0.14^b^ | 1.04±0.08^a^ | 0.010 |
| *sirt1* | 0.51±0.03 | 0.53±0.04 | 0.57±0.06 | 0.55±0.04 | 0.764 |
| *sirt2* | 2.01±0.13 | 1.80±0.15 | 1.81±0.06 | 1.77±0.16 | 0.598 |
| *sirt3* | 0.34±0.03 | 0.30±0.04 | 0.35±0.03 | 0.23±0.01 | 0.300 |
| *sirt4* | 0.14±0.01 | 0.13±0.01 | 0.13±0.01 | 0.13±0.01 | 0.738 |
| *sirt5* | 2.14±0.16 | 1.86±0.17 | 1.73±0.13 | 1.72±0.15 | 0.217 |
| *pgc1α* | 0.18±0.03 | 0.19±0.03 | 0.17±0.03 | 0.22±0.04 | 0.812 |
| *cpt1a* | 2.81±0.36 | 1.72±0.23 | 2.16±0.30 | 2.34±0.20 | 0.082 |
| *cs* | 6.50±0.48^a^ | 4.60±0.32^b^ | 5.81±0.34^ab^ | 4.91±0.35^b^ | 0.008 |
| *nd2* | 184.9±9.65 | 182.6±18.13 | 235.7±17.7 | 204.4±20.76 | 0.161 |
| *ndufaf2* | 2.42±0.15 | 2.39±0.11 | 2.32±0.15 | 2.31±0.13 | 0.922 |
| *coxi* | 406.8±22.1^a^ | 259.7±47.5^b^ | 366.0±31.3^ab^ | 328.1±22.7^ab^ | 0.028 |
| *sco1* | 0.49±0.06 | 0.43±0.05 | 0.44±0.02 | 0.35±0.03 | 0.136 |
| *ucp1* | 108.0±11.71 | 92.37±20.75 | 109.8±14.42 | 112.3±13.36 | 0.795 |
| *ucp2* | 0.02±0.01 | 0.01±0.01 | 0.01±0.01 | 0.01±0.01 | 0.300 |
| *lxrα* | 5.66±0.78^a^ | 3.34±0.33^b^ | 4.62±0.41^ab^ | 4.10±0.36^ab^ | 0.038 |
| *pparα* | 16.77±1.13 | 14.43±2.06 | 19.06±1.70 | 16.42±1.36 | 0.276 |
| *pparγ* | 5.50±0.57^a^ | 3.79±0.55^b^ | 3.95±0.17^b^ | 3.86±0.37^b^ | 0.037 |

^1^Result values from one-way analysis of variance
