# Supplementary material for: Somatotropic Axis Regulation Unravels the Differential Effects of Nutritional and Environmental Factors in Growth Performance of Marine Farmed Fishes
Source: Front Endocrinol (Lausanne). 2018 Nov 27;9:687. doi: 10.3389/fendo.2018.00687 (PMC6277588; doi:10.3389/fendo.2018.00687)
Supplement: Supplementary file 4 [file Table_4.DOCX]

**Supplementary Table S4.** Relative gene expression of growth-related genes in the skeletal muscle of gilthead sea bream sampled in August. Data are the mean±SEM of 6-7 fishes. All data are referenced to the expression level of *igf-iir* of control fishes (D1 diet) with an arbitrarily assigned value of 1. Different superscript letters in each row indicate significant differences among dietary treatments (P < 0.05; ANOVA followed by Student-Newman-Keuls test).

|  | D1 | D2 | D3 | D4 | P-value^1^ |
| --- | --- | --- | --- | --- | --- |
| *ghr-i* | 8.22±0.46 | 7.12±0.96 | 6.81±0.58 | 7.52±0.55 | 0.480 |
| *ghr-ii* | 3.63±0.37 | 2.60±0.37 | 2.96±0.76 | 2.55±0.50 | 0.501 |
| *igf-i* | 0.59±0.06 | 0.65±0.13 | 0.69±0.09 | 0.61±0.04 | 0.789 |
| *igf-ii* | 3.2±0.32 | 3.21±0.28 | 3.11±0.26 | 2.76±0.36 | 0.705 |
| *igfbp-1a* | 0.31±0.05 | 0.40±0.05 | 0.45±0.05 | 0.31±0.05 | 0.062 |
| *igfbp-3* | 10.42±1.55 | 7.59±1.02 | 11.22±1.55 | 7.30±0.44 | 0.051 |
| *igfbp-4* | 0.49±0.07 | 0.67±0.15 | 0.45±0.05 | 0.70±0.17 | 0.419 |
| *igfbp-5b* | 8.72±0.60 | 6.65±0.59 | 8.22±0.57 | 6.67±0.63 | 0.050 |
| *igfbp-6b* | 0.79±0.13 | 0.69±0.12 | 1.10±0.17 | 0.61±0.10 | 0.203 |
| *insr* | 1.44±0.09 | 1.21±0.07 | 1.42±0.09 | 1.31±0.16 | 0.451 |
| *igf-ira* | 1.41±0.12 | 1.17±0.12 | 1.27±0.07 | 1.17±0.13 | 0.384 |
| *igf-iir* | 0.83±0.13 | 0.65±0.03 | 0.60±0.02 | 0.61±0.08 | 0.189 |
| *myod1* | 15.23±1.23 | 12.22±0.72 | 16.74±1.16 | 15.07±1.70 | 0.059 |
| *myod2* | 8.04±1.16 | 7.02±1.01 | 8.75±1.18 | 6.83±0.78 | 0.517 |
| *myf5* | 1.05±0.09 | 1.07±0.06 | 1.12±0.05 | 0.97±0.08 | 0.544 |
| *myf6* | 1.03±0.09 | 0.96±0.07 | 1.23±0.04 | 1.08±0.22 | 0.530 |
| *mstn* | 5.21±1.35 | 4.51±0.45 | 6.17±1.82 | 5.70±0.96 | 0.493 |
| *mef2a* | 36.17±2.2 | 34.08±1.95 | 39.18±2.17 | 37.30±3.13 | 0.543 |
| *mef2c* | 8.77±0.44 | 7.80±0.42 | 8.55±0.51 | 8.09±0.57 | 0.523 |
| *fst* | 0.84±0.15 | 0.88±0.22 | 0.88±0.03 | 0.89±0.20 | 0.995 |
| *cav3* | 73.81±4.50 | 71.88±2.71 | 87.10±6.41 | 77.70±3.76 | 0.128 |
| *des* | 194.1±15.23 | 202.2±8.80 | 204.1±12.37 | 198.8±22.59 | 0.972 |
| *cdh15* | 1.62±0.10^a^ | 1.80±0.11^ab^ | 2.07±0.09^b^ | 1.65±0.12^a^ | 0.020 |
| *pcna* | 2.73±0.29 | 2.57±0.20 | 3.00±0.20 | 2.26±0.26 | 0.204 |
| *pax7* | 0.12±0.01 | 0.15±0.02 | 0.14±0.02 | 0.15±0.01 | 0.383 |
| *sox3* | 0.06±0.03 | 0.02±0.01 | 0.04±0.01 | 0.03±0.01 | 0.690 |
| *met* | 0.37±0.04 | 0.34±0.04 | 0.33±0.03 | 0.26±0.02 | 0.145 |
| *capn1* | 3.92±0.22 | 3.91±0.34 | 4.21±0.30 | 3.85±0.29 | 0.808 |
| *capn2* | 5.16±0.35 | 5.56±0.71 | 5.32±0.33 | 4.96±0.54 | 0.850 |
| *capn3* | 13.76±0.77^a^ | 13.45±0.86^a^ | 18.24±0.94^b^ | 12.13±1.51^a^ | 0.003 |
| *cast* | 13.91±1.15 | 11.21±0.50 | 14.24±1.83 | 12.36±0.87 | 0.312 |
| *ctsb* | 7.12±0.58 | 7.19±0.45 | 5.98±0.37 | 6.10±0.69 | 0.262 |
| *ctsd* | 0.91±0.07 | 0.96±0.06 | 0.86±0.05 | 0.73±0.09 | 0.151 |
| *ctsl* | 11.36±0.90 | 11.32±1.19 | 10.78±0.65 | 10.22±0.79 | 0.776 |
| *ctss* | 2.04±0.27 | 2.32±0.24 | 1.74±0.30 | 1.57±0.23 | 0.224 |
| *psmd4* | 1.97±0.10 | 1.92±0.05 | 2.11±0.08 | 1.75±0.17 | 0.179 |
| *psd12* | 5.95±0.40 | 5.85±0.50 | 6.71±0.30 | 5.12±0.53 | 0.105 |
| *psma5* | 3.10±0.23^ab^ | 2.88±0.19^ab^ | 3.52±0.20^b^ | 2.41±0.31^a^ | 0.023 |
| *psmb1a* | 7.85±0.57^ab^ | 7.59±0.20^ab^ | 9.04±0.36^b^ | 6.72±0.69^a^ | 0.028 |
| *uchl3* | 4.02±0.19^a^ | 3.79±0.07^ab^ | 4.23±0.30^a^ | 3.15±0.26^b^ | 0.013 |
| *ube2a* | 4.29±0.86 | 3.46±0.14 | 3.69±0.35 | 3.06±0.27 | 0.389 |

**Supplementary Table S4. (continued)**

|  | D1 | D2 | D3 | D4 | P-value^1^ |
| --- | --- | --- | --- | --- | --- |
| *ube2d2* | 1.98±0.19 | 1.92±0.07 | 2.16±0.17 | 1.61±0.12 | 0.091 |
| *ube2l3* | 17.42±1.00 | 15.68±1.02 | 18.37±1.15 | 15.48±0.82 | 0.153 |
| *ube2n* | 13.03±0.88^ab^ | 12.14±0.30^ab^ | 14.60±1.05^b^ | 10.57±1.07^a^ | 0.025 |
| *cul2* | 2.05±0.16 | 1.84±0.04 | 2.15±0.08 | 1.75±0.11 | 0.051 |
| *cul3* | 3.02±0.15 | 2.65±0.14 | 3.27±0.12 | 2.72±0.25 | 0.076 |
| *cul5* | 0.68±0.07 | 0.67±0.04 | 0.81±0.11 | 0.60±0.06 | 0.228 |
| *mthsp10* | 5.39±0.79 | 5.60±0.63 | 6.83±0.84 | 4.86±0.67 | 0.300 |
| *hsp30* | 0.08±0.06 | 0.08±0.02 | 0.11±0.07 | 0.08±0.04 | 0.966 |
| *mthsp60* | 2.10±0.19 | 2.17±0.19 | 2.58±0.22 | 1.85±0.20 | 0.110 |
| *mthsp70* | 4.44±0.38 | 4.21±0.30 | 5.40±0.49 | 4.48±0.38 | 0.197 |
| *hsp90α* | 60.02±8.51 | 69.10±8.46 | 73.07±8.37 | 57.80±3.82 | 0.434 |
| *hsp90β* | 29.87±2.55 | 31.26±2.86 | 31.34±2.05 | 27.65±2.45 | 0.690 |
| *grp-170* | 1.83±0.15 | 1.81±0.13 | 1.98±0.09 | 1.60±0.16 | 0.282 |
| *grp-94* | 3.23±0.29 | 3.04±0.16 | 2.96±0.26 | 2.41±0.14 | 0.085 |
| *der-1* | 9.18±0.55 | 9.48±0.37 | 10.84±0.67 | 8.76±0.60 | 0.079 |
| *il-1β* | 0.05±0.01 | 0.04±0.01 | 0.05±0.01 | 0.05±0.01 | 0.659 |
| *il-1r1* | 0.61±0.04 | 0.62±0.09 | 0.55±0.06 | 0.47±0.07 | 0.367 |
| *il-1r2* | 0.02±0.01 | 0.02±0.01 | 0.01±0.01 | 0.01±0.01 | 0.086 |
| *il-6* | 0.01±0.01 | 0.01±0.01 | 0.01±0.01 | 0.01±0.01 | 0.456 |
| *il-6ra* | 0.44±0.03 | 0.44±0.07 | 0.47±0.02 | 0.38±0.06 | 0.596 |
| *il-6rb* | 2.91±0.16 | 2.87±0.12 | 3.01±0.21 | 2.40±0.22 | 0.122 |
| *il-8* | 0.04±0.01 | 0.04±0.01 | 0.04±0.01 | 0.04±0.01 | 0.787 |
| *il-10* | 0.05±0.01 | 0.06±0.01 | 0.05±0.01 | 0.04±0.01 | 0.356 |
| *il-10ra* | 0.06±0.01 | 0.06±0.01 | 0.05±0.01 | 0.04±0.01 | 0.438 |
| *il-10rb* | 0.73±0.05 | 0.91±0.05 | 0.76±0.04 | 0.73±0.08 | 0.125 |
| *tnfα* | 0.10±0.01^ab^ | 0.10±0.01^ab^ | 0.12±0.01^b^ | 0.08±0.01^a^ | 0.025 |
| *tradd* | 0.29±0.02 | 0.32±0.03 | 0.34±0.02 | 0.27±0.03 | 0.251 |
| *sirt1* | 0.61±0.04 | 0.61±0.02 | 0.61±0.02 | 0.55±0.06 | 0.672 |
| *sirt2* | 1.27±0.11 | 1.19±0.04 | 1.26±0.07 | 1.06±0.11 | 0.314 |
| *sirt3* | 0.17±0.01 | 0.17±0.02 | 0.19±0.02 | 0.15±0.01 | 0.310 |
| *sirt4* | 0.14±0.01 | 0.12±0.01 | 0.14±0.01 | 0.12±0.01 | 0.254 |
| *sirt5* | 1.89±0.15 | 1.74±0.13 | 1.95±0.08 | 1.64±0.12 | 0.282 |
| *pgc1α* | 0.32±0.09 | 0.24±0.08 | 0.28±0.06 | 0.32±0.10 | 0.896 |
| *cpt1a* | 5.66±0.59 | 5.16±0.66 | 4.50±0.36 | 6.52±0.99 | 0.226 |
| *cs* | 42.9±3.49 | 38.41±2.72 | 40.39±2.24 | 39.05±3.97 | 0.769 |
| *nd2* | 182.3±24.2 | 224.6±11.9 | 231.1±26.5 | 205.0±14.1 | 0.354 |
| *ndufaf2* | 2.09±0.15 | 2.00±0.11 | 2.04±0.14 | 1.80±0.15 | 0.462 |
| *coxi* | 553.6±72.1 | 503.9±22.8 | 551.4±24.6 | 503.8±11.4 | 0.711 |
| *sco1* | 0.32±0.03 | 0.29±0.02 | 0.31±0.03 | 0.33±0.04 | 0.812 |
| *ucp2* | 0.64±0.10 | 0.61±0.09 | 0.91±0.18 | 0.72±0.16 | 0.365 |
| *ucp3* | 14.36±2.62 | 13.30±3.07 | 20.36±2.42 | 16.72±3.42 | 0.363 |
| *lxrα* | 0.72±0.05 | 0.70±0.02 | 0.65±0.04 | 0.60±0.04 | 0.137 |
| *pparα* | 1.98±0.10^a^ | 1.80±0.12^ab^ | 1.64±0.11^ab^ | 1.37±0.16^b^ | 0.013 |
| *pparγ* | 1.64±0.14^a^ | 1.62±0.48^ab^ | 1.32±0.24^ab^ | 1.10±0.10^b^ | 0.015 |

^1^Result values from one-way analysis of variance
