# Supplementary material for: Somatotropic Axis Regulation Unravels the Differential Effects of Nutritional and Environmental Factors in Growth Performance of Marine Farmed Fishes
Source: Front Endocrinol (Lausanne). 2018 Nov 27;9:687. doi: 10.3389/fendo.2018.00687 (PMC6277588; doi:10.3389/fendo.2018.00687)
Supplement: Supplementary file 5 [file Table_5.DOC]

**Supplementary Table S5.** Relative gene expression of growth-related genes in the liver of gilthead sea bream sampled in December. Data are the mean±SEM of 6-7 fishes. All data are referenced to the expression level of *igf-iir* of control fishes (D1 diet) with an arbitrarily assigned value of 1. Different superscript letters in each row indicate significant differences among dietary treatments (P < 0.05; ANOVA followed by Student-Newman-Keuls test).

|  | D1 | D2 | D3 | D4 | P-value^1^ |
| --- | --- | --- | --- | --- | --- |
| *ghr-i* | 7.47±0.81 | 6.55±1.02 | 7.24±1.06 | 5.14±0.51 | 0.267 |
| *ghr-ii* | 4.60±0.51^a^ | 4.01±0.30^ab^ | 3.84±0.64^ab^ | 2.41±0.39^b^ | 0.023 |
| *igf-i* | 35.65±6.48 | 32.14±4.18 | 29.33±2.20 | 22.57±2.49 | 0.157 |
| *igf-ii* | 15.09±2.76 | 13.02±1.96 | 14.89±1.43 | 10.26±0.65 | 0.147 |
| *igfbp-1a* | 0.12±0.01 | 0.11±0.01 | 0.11±0.01 | 0.09±0.01 | 0.010 |
| *igfbp-2b* | 5.33±0.46 | 4.99±0.53 | 5.68±0.36 | 4.03±0.18 | 0.051 |
| *igfbp-4* | 3.20±0.34^ab^ | 3.39±0.24^a^ | 2.72±0.26^ab^ | 2.17±0.17^b^ | 0.016 |
| *igfbp-5b* | 1.22±0.19 | 1.36±0.30 | 1.20±0.10 | 1.07±0.12 | 0.785 |
| *igfals* | 114.1±8.51^ab^ | 145.1±9.81^a^ | 126.9±10.99^ab^ | 102.3±8.24^b^ | 0.024 |
| *insr* | 2.18±0.101 | 2.34±0.22 | 2.36±0.13 | 1.87±0.11 | 0.105 |
| *igf-ira* | 0.19±0.02 | 0.19±0.02 | 0.23±0.03 | 0.17±0.02 | 0.377 |
| *igf-iir* | 1.02±0.08 | 1.06±0.09 | 1.23±0.06 | 0.96±0.10 | 0.141 |
| *mef2a* | 1.38±0.08 | 1.40±0.09 | 1.64±0.13 | 1.39±0.21 | 0.496 |
| *mef2c* | 0.12±0.01 | 0.10±0.01 | 0.12±0.01 | 0.10±0.01 | 0.469 |
| *vim* | 0.24±0.02 | 0.20±0.03 | 0.27±0.03 | 0.20±0.02 | 0.258 |
| *pcna* | 2.10±0.27 | 2.39±0.33 | 1.41±0.12 | 2.48±0.50 | 0.130 |
| *met* | 2.85±0.17 | 3.30±0.35 | 2.92±0.18 | 2.37±0.31 | 0.135 |
| *capn1* | 1.04±0.08 | 0.90±0.05 | 1.00±0.07 | 0.81±0.07 | 0.101 |
| *capn2* | 0.77±0.13 | 0.81±0.07 | 1.15±0.09 | 0.89±0.12 | 0.079 |
| *capn3* | 0.06±0.01 | 0.05±0.01 | 0.07±0.01 | 0.06±0.01 | 0.245 |
| *cast* | 2.79±0.33 | 2.69±0.20 | 3.39±0.18 | 2.84±0.26 | 0.222 |
| *ctsb* | 9.58±0.77 | 10.62±1.14 | 11.89±0.97 | 8.61±0.88 | 0.115 |
| *ctsd* | 9.12±1.64^a^ | 7.01±0.87^ab^ | 6.37±1.11^ab^ | 4.06±0.50^b^ | 0.030 |
| *ctsl* | 55.28±8.39 | 51.77±5.13 | 60.68±3.96 | 47.39±5.37 | 0.458 |
| *ctss* | 1.03±0.10 | 0.96±0.12 | 1.28±0.11 | 1.19±0.08 | 0.224 |
| *psmd4* | 1.45±0.13 | 1.65±0.19 | 1.41±0.16 | 1.37±0.17 | 0.651 |
| *psd12* | 2.79±0.29 | 3.60±0.53 | 2.43±0.27 | 2.70±0.23 | 0.127 |
| *psma5* | 2.50±0.33 | 3.18±0.42 | 2.48±0.38 | 2.81±0.34 | 0.511 |
| *psmb1a* | 4.75±0.43 | 6.02±0.72 | 5.07±0.54 | 5.62±0.75 | 0.499 |
| *uchl3* | 2.64±0.62 | 2.79±0.46 | 2.26±0.34 | 2.85±0.40 | 0.830 |
| *ube2a* | 1.32±0.07 | 1.47±0.14 | 1.33±0.10 | 1.10±0.09 | 0.102 |
| *ube2d2* | 3.64±0.21^ab^ | 4.52±0.38^a^ | 3.57±0.16^ab^ | 3.37±0.22^b^ | 0.019 |
| *ube2l3* | 8.20±0.43 | 9.03±0.73 | 8.10±0.54 | 7.78±0.73 | 0.542 |
| *ube2n* | 2.80±0.32 | 3.53±0.34 | 2.83±0.19 | 3.23±0.34 | 0.288 |
| *cul2* | 0.81±0.05 | 0.88±0.07 | 0.82±0.05 | 0.79±0.07 | 0.734 |
| *cul3* | 0.81±0.05 | 0.82±0.06 | 0.76±0.06 | 0.65±0.04 | 0.103 |
| *cul5* | 0.32±0.03 | 0.37±0.03 | 0.36±0.02 | 0.31±0.02 | 0.364 |
| *mthsp10* | 7.12±0.40 | 9.20±1.76 | 6.24±0.74 | 8.02±0.83 | 0.255 |
| *mthsp30* | 0.03±0.00 | 0.03±0.00 | 0.03±0.01 | 0.02±0.00 | 0.307 |
| *mthsp60* | 2.17±0.24 | 3.29±0.68 | 1.96±0.23 | 2.43±0.22 | 0.094 |
| *mthsp70* | 3.19±0.51 | 4.18±0.50 | 3.04±0.20 | 3.32±0.48 | 0.283 |

**Supplementary Table S5.** **(continued)**

|  | R1 | R2 | R3 | R4 | P-value^1^ |
| --- | --- | --- | --- | --- | --- |
| *hsp90β* | 95.88±4.96 | 86.63±6.16 | 97.60±5.23 | 84.06±9.03 | 0.389 |
| *grp-170* | 12.67±1.56 | 13.37±1.70 | 11.52±1.11 | 11.75±0.58 | 0.740 |
| *grp-94* | 13.18±1.69 | 19.54±3.76 | 11.83±2.28 | 14.43±1.63 | 0.160 |
| *der-1* | 8.93±0.88 | 9.92±1.05 | 8.15±0.58 | 8.26±0.59 | 0.367 |
| *il-1β* | 0.01±0.00 | 0.01±0.00 | 0.01±0.00 | 0.01±0.00 | 0.171 |
| *il-1r1* | 1.17±0.14 | 1.25±0.18 | 1.09±0.04 | 0.92±0.11 | 0.343 |
| *il-1r2* | 0.01±0.00 | 0.01±0.00 | 0.02±0.00 | 0.01±0.00 | 0.074 |
| *il-6ra* | 4.26±0.21 | 4.06±0.36 | 4.74±0.26 | 3.69±0.40 | 0.153 |
| *il-6rb* | 2.71±0.15 | 3.12±0.25 | 2.93±0.23 | 2.35±0.14 | 0.063 |
| *il-8* | 0.01±0.00 | 0.01±0.00 | 0.01±0.00 | 0.01±0.00 | 0.182 |
| *il-8ra* | 0.04±0.01 | 0.05±0.01 | 0.07±0.01 | 0.05±0.01 | 0.448 |
| *il-10* | 0.02±0.00 | 0.02±0.00 | 0.02±0.00 | 0.02±0.00 | 0.389 |
| *il-10ra* | 0.06±0.01^b^ | 0.05±0.00^b^ | 0.09±0.01^a^ | 0.06±0.01^b^ | 0.001 |
| *il-10rb* | 2.71±0.23 | 3.01±0.35 | 3.17±0.23 | 2.50±0.24 | 0.305 |
| *tnf-α* | 0.05±0.01 | 0.04±0.00 | 0.05±0.01 | 0.03±0.01 | 0.088 |
| *tradd* | 0.73±0.05 | 0.73±0.06 | 0.75±0.06 | 0.69±0.05 | 0.899 |
| *sirt1* | 0.43±0.03 | 0.41±0.04 | 0.48±0.03 | 0.35±0.03 | 0.083 |
| *sirt2* | 0.91±0.06 | 0.88±0.05 | 0.91±0.05 | 0.71±0.05 | 0.063 |
| *sirt3* | 0.20±0.02 | 0.20±0.03 | 0.16±0.01 | 0.19±0.02 | 0.575 |
| *sirt4* | 0.08±0.01 | 0.07±0.01 | 0.09±0.01 | 0.06±0.00 | 0.242 |
| *sirt5* | 1.14±0.10 | 1.29±0.14 | 1.18±0.07 | 0.98±0.07 | 0.213 |
| *pgc1α* | 0.25±0.02 | 0.17±0.03 | 0.19±0.02 | 0.18±0.02 | 0.071 |
| *cpt1a* | 3.46±0.42 | 3.40±0.34 | 3.74±0.27 | 3.70±0.40 | 0.880 |
| *cs* | 3.91±0.28 | 3.65±0.24 | 3.62±0.22 | 3.09±0.25 | 0.156 |
| *nd2* | 80.07±7.44 | 133.24±23.26 | 103.37±26.43 | 79.60±8.94 | 0.146 |
| *ndufaf2* | 1.84±0.05 | 1.75±0.16 | 1.80±0.15 | 1.40±0.10 | 0.066 |
| *coxi* | 175.9±14.86^a^ | 124.6±6.64^bc^ | 162.2±17.99^ab^ | 107.5±8.00^c^ | 0.003 |
| *sco1* | 0.36±0.04 | 0.39±0.04 | 0.36±0.02 | 0.30±0.04 | 0.342 |
| *ucp1* | 30.36±3.22 | 28.29±2.46 | 28.27±2.66 | 22.42±2.25 | 0.208 |
| *ucp2* | 0.01±0.00 | 0.01±0.00 | 0.01±0.00 | 0.01±0.00 | 0.431 |
| *lxrα* | 3.32±0.17 | 3.25±0.20 | 3.72±0.21 | 3.26±0.23 | 0.326 |
| *pparα* | 9.63±0.77^a^ | 7.45±1.12^ab^ | 8.09±0.52^ab^ | 5.78±0.26^b^ | 0.011 |
| *pparγ* | 3.10±0.35 | 2.40±0.20 | 2.86±0.13 | 2.73±0.32 | 0.325 |

^1^Result values from one-way analysis of variance
