# Supplementary material for: Somatotropic Axis Regulation Unravels the Differential Effects of Nutritional and Environmental Factors in Growth Performance of Marine Farmed Fishes
Source: Front Endocrinol (Lausanne). 2018 Nov 27;9:687. doi: 10.3389/fendo.2018.00687 (PMC6277588; doi:10.3389/fendo.2018.00687)
Supplement: Supplementary file 6 [file Table_6.DOC]

**Supplementary Table S6.** Relative gene expression of growth-related genes in the skeletal muscle of gilthead sea bream sampled in December. Data are the mean±SEM of 6-7 fishes. All data are referenced to the expression level of *igf-iir* of control fishes (D1 diet) with an arbitrarily assigned value of 1. Different superscript letters in each row indicate significant differences among dietary treatments (P < 0.05; ANOVA followed by Student-Newman-Keuls test).

|  | D1 | D2 | D3 | D4 | P-value^1^ |
| --- | --- | --- | --- | --- | --- |
| *ghr-i* | 3.89±0.44 | 3.15±0.27 | 3.58±0.27 | 3.26±0.28 | 0.391 |
| *ghr-ii* | 0.73±0.11 | 0.56±0.11 | 0.59±0.11 | 0.53±0.10 | 0.601 |
| *igf-i* | 0.11±0.04 | 0.12±0.02 | 0.15±0.03 | 0.12±0.02 | 0.829 |
| *igf-ii* | 0.92±0.10 | 0.88±0.07 | 1.08±0.07 | 1.01±0.09 | 0.355 |
| *igfbp-1a* | 0.14±0.01 | 0.12±0.01 | 0.13±0.01 | 0.12±0.01 | 0.534 |
| *igfbp-3* | 1.34±0.17 | 1.08±0.10 | 1.00±0.07 | 1.19±0.11 | 0.268 |
| *igfbp-4* | 0.16±0.04 | 0.25±0.07 | 0.23±0.04 | 0.22±0.04 | 0.682 |
| *igfbp-5b* | 2.18±0.37 | 2.85±0.17 | 3.00±0.31 | 3.10±0.30 | 0.154 |
| *igfbp-6b* | 0.14±0.03 | 0.11±0.01 | 0.13±0.02 | 0.17±0.01 | 0.713 |
| *insr* | 0.90±0.07 | 0.83±0.06 | 1.00±0.07 | 1.04±0.07 | 0.149 |
| *igf-ira* | 1.13±0.08 | 0.92±0.10 | 1.16±0.05 | 1.10±0.11 | 0.279 |
| *igf-iir* | 0.99±0.13 | 0.82±0.10 | 0.96±0.06 | 0.97±0.08 | 0.616 |
| *myod1* | 7.48±0.73 | 6.97±0.33 | 7.82±0.72 | 6.83±0.70 | 0.694 |
| *myod2* | 2.28±0.39 | 2.13±0.24 | 2.41±0.19 | 2.06±0.28 | 0.804 |
| *myf5* | 0.23±0.02 | 0.24±0.02 | 0.26±0.01 | 0.26±0.02 | 0.637 |
| *myf6* | 0.36±0.05 | 0.31±0.03 | 0.36±0.03 | 0.33±0.03 | 0.746 |
| *mstn* | 2.33±0.47 | 3.16±0.64 | 3.31±0.38 | 3.58±0.47 | 0.389 |
| *mef2a* | 31.88±5.23 | 28.60±2.41 | 36.44±4.60 | 32.46±4.74 | 0.698 |
| *mef2c* | 5.51±0.64 | 5.65±0.37 | 6.37±0.40 | 6.22±0.55 | 0.567 |
| *fst* | 0.18±0.03 | 0.12±0.02 | 0.18±0.02 | 0.18±0.02 | 0.232 |
| *cav3* | 18.10±1.68 | 14.61±0.64 | 16.78±0.95 | 16.61±1.20 | 0.308 |
| *des* | 82.77±7.92 | 66.60±6.84 | 85.75±5.37 | 79.30±6.80 | 0.266 |
| *vim* | 0.22±0.03 | 0.23±0.01 | 0.30±0.02 | 0.27±0.03 | 0.141 |
| *cdh15* | 0.44±0.08 | 0.51±0.05 | 0.46±0.02 | 0.53±0.07 | 0.583 |
| *pcna* | 0.43±0.04 | 0.39±0.03 | 0.46±0.03 | 0.49±0.08 | 0.595 |
| *pax7* | 0.10±0.02 | 0.05±0.01 | 0.07±0.01 | 0.07±0.01 | 0.134 |
| *sox3* | 0.00±0.00 | 0.00±0.00 | 0.03±0.01 | 0.02±0.01 | 0.468 |
| *met* | 0.09±0.01 | 0.08±0.01 | 0.10±0.01 | 0.09±0.01 | 0.334 |
| *capn1* | 0.78±0.10 | 0.70±0.04 | 0.95±0.04 | 0.90±0.08 | 0.090 |
| *capn2* | 0.71±0.10 | 0.81±0.06 | 1.13±0.17 | 1.19±0.22 | 0.128 |
| *capn3* | 3.26±0.61 | 3.12±0.37 | 4.84±0.52 | 4.55±0.75 | 0.129 |
| *cast* | 8.42±1.32 | 7.01±0.75 | 8.77±0.95 | 8.75±0.86 | 0.610 |
| *ctsb* | 1.89±0.15 | 1.77±0.14 | 2.28±0.14 | 2.17±0.17 | 0.102 |
| *ctsd* | 0.32±0.05 | 0.29±0.02 | 0.40±0.02 | 0.35±0.04 | 0.194 |
| *ctsl* | 4.55±0.68 | 3.96±0.45 | 4.83±0.23 | 4.36±0.47 | 0.649 |
| *ctss* | 0.21±0.02 | 0.23±0.04 | 0.32±0.04 | 0.31±0.06 | 0.218 |
| *psmd4* | 0.83±0.10 | 0.80±0.06 | 0.95±0.05 | 0.91±0.11 | 0.622 |
| *psd12* | 3.12±0.43 | 3.25±0.31 | 3.46±0.28 | 3.54±0.28 | 0.789 |
| *psma5* | 1.62±0.21 | 1.66±0.14 | 2.00±0.12 | 2.01±0.17 | 0.202 |
| *psmb1a* | 3.36±0.57 | 3.16±0.25 | 3.68±0.24 | 3.62±0.36 | 0.763 |
| *uchl3* | 0.76±0.12 | 0.76±0.06 | 0.90±0.04 | 0.79±0.09 | 0.624 |
| *ube2a* | 1.73±0.22 | 1.66±0.16 | 1.91±0.10 | 1.71±0.16 | 0.703 |

**Supplementary Table S6. (continued)**

|  | D1 | D2 | D3 | D4 | P-value^1^ |
| --- | --- | --- | --- | --- | --- |
| *ube2d2* | 0.69±0.07 | 0.82±0.09 | 0.87±0.04 | 0.82±0.05 | 0.292 |
| *ube2l3* | 5.56±0.59 | 5.28±0.33 | 5.83±0.33 | 5.94±0.42 | 0.725 |
| *ube2n* | 3.46±0.48 | 3.55±0.20 | 4.26±0.22 | 4.02±0.42 | 0.359 |
| *cul2* | 0.68±0.09 | 0.70±0.05 | 0.77±0.04 | 0.75±0.07 | 0.726 |
| *cul3* | 1.63±0.22 | 1.51±0.08 | 1.65±0.12 | 1.64±0.14 | 0.913 |
| *cul5* | 0.16±0.02 | 0.17±0.01 | 0.19±0.01 | 0.19±0.02 | 0.410 |
| *mthsp10* | 1.09±0.22 | 1.14±0.14 | 1.66±0.20 | 1.27±0.18 | 0.153 |
| *mthsp30* | 0.00±0.00 | 0.00±0.00 | 0.01±0.00 | 0.04±0.04 | 0.455 |
| *mthsp60* | 0.69±0.12 | 0.62±0.06 | 0.83±0.11 | 0.71±0.08 | 0.469 |
| *mthsp70* | 3.11±0.34 | 2.90±0.28 | 3.41±0.20 | 3.42±0.24 | 0.480 |
| *hsp90α* | 23.29±4.60 | 22.46±2.96 | 23.32±2.01 | 32.08±4.29 | 0.205 |
| *hsp90β* | 7.14±1.11 | 6.06±0.25 | 8.90±0.66 | 9.22±1.15 | 0.083 |
| *grp-170* | 0.92±0.10 | 0.90±0.05 | 0.99±0.09 | 0.98±0.06 | 0.852 |
| *grp-94* | 0.89±0.11 | 0.89±0.04 | 0.89±0.05 | 0.99±0.11 | 0.789 |
| *der-1* | 4.77±0.47 | 4.78±0.32 | 5.40±0.21 | 4.85±0.27 | 0.443 |
| *il-1β* | 0.01±0.00 | 0.01±0.00 | 0.01±0.00 | 0.01±0.00 | 0.754 |
| *il-1r1* | 0.07±0.01 | 0.07±0.00 | 0.10±0.01 | 0.10±0.02 | 0.350 |
| *il-1r2* | 0.00±0.00 | 0.00±0.00 | 0.01±0.00 | 0.00±0.00 | 0.299 |
| *il-6* | 0.00±0.00 | 0.00±0.00 | 0.00±0.00 | 0.00±0.00 | 0.309 |
| *il-6ra* | 0.10±0.01^a^ | 0.11±0.01^ab^ | 0.15±0.01^b^ | 0.14±0.01^ab^ | 0.010 |
| *il-6rb* | 0.87±0.09 | 0.85±0.05 | 1.02±0.04 | 0.97±0.07 | 0.227 |
| *il-8* | 0.00±0.00 | 0.00±0.00 | 0.00±0.00 | 0.01±0.00 | 0.108 |
| *il-10* | 0.01±0.00 | 0.01±0.00 | 0.01±0.00 | 0.01±0.00 | 0.173 |
| *il-10ra* | 0.01±0.00 | 0.01±0.00 | 0.01±0.00 | 0.01±0.00 | 0.606 |
| *il-10rb* | 0.32±0.05 | 0.31±0.01 | 0.36±0.02 | 0.34±0.03 | 0.599 |
| *tnf-α* | 0.01±0.00 | 0.01±0.00 | 0.01±0.00 | 0.01±0.00 | 0.640 |
| *tradd* | 0.11±0.01 | 0.10±0.00 | 0.13±0.00 | 0.13±0.01 | 0.083 |
| *sirt1* | 0.31±0.03 | 0.27±0.02 | 0.35±0.02 | 0.35±0.02 | 0.128 |
| *sirt2* | 0.31±0.04 | 0.29±0.03 | 0.35±0.03 | 0.30±0.02 | 0.634 |
| *sirt3* | 0.05±0.01 | 0.04±0.00 | 0.05±0.00 | 0.06±0.01 | 0.414 |
| *sirt4* | 0.04±0.00 | 0.04±0.00 | 0.05±0.00 | 0.04±0.01 | 0.672 |
| *sirt5* | 0.91±0.12 | 0.81±0.15 | 1.02±0.11 | 1.01±0.14 | 0.675 |
| *pgc1α* | 0.19±0.05 | 0.11±0.02 | 0.31±0.10 | 0.24±0.07 | 0.254 |
| *cpt1a* | 6.51±1.02 | 6.02±0.45 | 8.15±0.53 | 7.50±0.72 | 0.192 |
| *cs* | 23.57±2.65 | 20.76±1.17 | 23.80±1.74 | 22.94±2.00 | 0.738 |
| *nd2* | 78.17±10.37 | 67.56±7.29 | 79.09±7.09 | 74.45±6.07 | 0.750 |
| *ndufaf2* | 1.82±0.20 | 1.55±0.10 | 1.86±0.11 | 1.83±0.17 | 0.518 |
| *coxi* | 243.73±34.86 | 186.33±16.49 | 286.58±23.15 | 268.58±24.41 | 0.113 |
| *sco1* | 0.22±0.02 | 0.18±0.02 | 0.20±0.02 | 0.21±0.02 | 0.581 |
| *ucp2* | 0.04±0.01 | 0.04±0.00 | 0.05±0.00 | 0.05±0.01 | 0.366 |
| *ucp3* | 7.52±0.86 | 6.01±0.77 | 9.93±1.12 | 9.73±1.38 | 0.070 |
| *lxrα* | 0.30±0.04 | 0.30±0.03 | 0.39±0.03 | 0.34±0.03 | 0.230 |
| *pparα* | 0.49±0.04 | 0.35±0.04 | 0.61±0.09 | 0.42±0.05 | 0.054 |
| *pparγ* | 0.38±0.11 | 0.31±0.06 | 0.46±0.09 | 0.40±0.05 | 0.638 |

^1^Result values from one-way analysis of variance
